# Reformulating the meta-analytical random effects model of the standardized mean difference as a mixture model

**DOI:** 10.3758/s13428-024-02554-6

**Published:** 2025-01-24

**Authors:** Manuel Suero, Juan Botella, Juan I. Duran, Desirée Blazquez-Rincón

**Affiliations:** 1 Facultad de Psicología, Universidad Autónoma de Madrid, Campus de Cantoblanco, C/ Ivan Pavlov, 6, 28049 Madrid, Spain; 2 Universidad a Distancia de Madrid, Madrid, Spain

**Keywords:** Random effects model, Meta-analysis, Mixture models, Standardized mean difference

## Abstract

The classical meta-analytical random effects model (REM) has some weaknesses when applied to the standardized mean difference, *g*. Essentially, the variance of the studies involved is taken as the conditional variance, given a *δ* value, instead of the unconditional variance. As a consequence, the estimators of the variances involve a dependency between the *g* values and their variances that distorts the estimates. The classical REM is expressed as a linear model and the variance of *g* is obtained through a framework of components of variance. Although the weaknesses of the REM are negligible in practical terms in a wide range of realistic scenarios, all together, they make up an approximate, simplified version of the meta-analytical random effects model. We present an alternative formulation, as a mixture model, and provide formulas for the expected value, variance and skewness of the marginal distribution of *g*. A Monte Carlo simulation supports the accuracy of the formulas. Then, unbiased estimators of both the mean and the variance of the true effects are proposed, and assessed through Monte Carlo simulations. The advantages of the mixture model formulation over the “classical” formulation are discussed.

**Supplementary Information:**

The online version contains supplementary material available at 10.3758/s13428-024-02554-6.

## Introduction

A primary goal in meta-analysis is to model the *k* independent values observed in an effect size (ES) index. Meta-analysts commonly use a statistical model with antecedents traced back mainly to Cochran (1954). Subsequently, Hedges (1982, 1983; Hedges & Olkin, 1985) proposed the so-called random effects model (REM), in which the ES index is expressed as a linear model. However, how REM is usually applied in practical contexts to ES indices with a specific characteristic has a fundamental flaw. We refer to cases in which the variance of the ES for a given true effect depends on the parameter, as for example, the standardized mean difference (SMD; Cohen, 1988; see Appendix A), usually represented as *g*. In this article, we refer only to *g*, although many of the arguments can be extended to other indices where the variance also depends on the parameter. The fundamental flaw consists of taking the conditional (to the parameter) variance of *g*, instead of the unconditional variance, to estimate the variance of *g* in the specific studies involved. Of course, this problem could only appear when the conditional and unconditional variances are different. Since the work of Hedges and his colleagues, many authors have highlighted this shortcoming, as well as other problems derived from this framework. Sometimes, the choice between conditional and unconditional variance can be seen as a choice between two suitable options. However, the conditional variance is a questionable choice, as we will see later. Furthermore, the modifications proposed to the REM for solving the problems have not challenged the general framework. Specifically, the linear model framework is maintained for *g*, obtaining its variance from a logic of components of variance. Essentially, many authors are well aware of the flaw, but consider the consequences to be negligible. We believe that some of the problems of the REM can be solved with a different formulation that allows deriving estimators from the moments-generating function of *g*.

In this article, we describe an alternative to the linear REM for *g*, formulated as a mixture model (MM), which does not include some of the problems of the classic REM. The literature includes precedents of MM for meta-analyzing several ES indices, as the Pearson correlation coefficient (Thomas, 1989). Notably, Böhning (2000) made an explicit proposal for mixture models as an alternative to the REM that is not restricted to the case of correlation coefficients. Based on this proposal, Malzahn et al. (2000) derived a “nonparametric” estimator of the variance of parametric effects, a framework later extended to the meta-analysis of the accuracy of diagnostic tools (Doebler & Holling, 2015; Holling et al., 2012).

Böhning’s (2000) formulation of the REM as an MM did not seem to attract the attention it deserves. The objective of his article was to promote the development of the MM for meta-analysis, as an alternative to the REM for *g* because it is exact (not approximated or simplified), very flexible, and does not incur the ambiguities or difficulties of the classic REM. Furthermore, it can be directly applied to ES indices whose variance depends on the parameter as well as to those whose variance does not depend on it. We have derived formulas for the probability density function of *g* and a general expression that allows derivation of the moment of order *r*. These formulas allow for the obtained formulas for the expected value, the variance, and the skewness of the distribution of *g*, avoiding the components of variance logic. Then, new estimators of the variance of true effects, or specific variance (*τ*^2^), and of the mean effect (*μ*_*Δ*_) are proposed.

We begin by describing the analytical scenario that is intended to be modeled, which is prototypical in meta-analysis. In "The practical problems of the random effects model with g" we explain the fundamental flaw and other problems of the REM, when applied to *g*. In "Introduction to mixture models" we make a brief general introduction to MM. In the central part of the article, "Reformulating the REM for SMD as a mixture model", we demonstrate how mixture models can be applied to the meta-analytic scenario with *g*. "Checking the accuracy of the formulas" provides some results on the accuracy of the formulas, while in "Estimating the specific variance by the method of the moments" we propose estimators of the mean and the variance of the true effects (*μ*_*Δ*_ and *τ*^2^), and assess its performance. Finally, we discuss the benefits of the formulation of the random effects as a MM.

## Modeling the meta-analytic scenario with the random effects model

In social sciences, it is assumed by default that the parameter that describes the effect under study varies among the studies that estimate the same nominal effect (Borenstein et al., 2010). Thus, not all educational interventions categorized under the label “self-assessment” nor all psychological interventions identified with the label “acceptance and commitment therapy” have the same true or parametric effect. The goal of the meta-analyst is to develop and fit a statistical model that recognizes that the primary studies provide statistical estimates of the respective parameters (or true effects), but those true effects vary from study to study. Sometimes part of that variation can be explained by study-level moderators. However, the most realistic assumption is that there is also some genuine, residual heterogeneity among the studies within the *class* encompassed by the label we use to designate them.

The REM is formulated by stating that the value of the ES index, *y*, for a particular study is an estimate of the specific parameter, *θ*, of that study. That is, if the true effect in a study is *θ*, the observed value of *y* can be expressed as:

1 $${y}_{i}=\theta +{e}_{i}$$

where *e*_*i*_ is the measurement error of *y*_*i*_ with respect to *θ*. However, the value of *θ* in the studies is itself a random variable with expected value *μ*_*Θ*_ and variance *τ*^2^. Being *θ*_*i*_ the true effect of study *i*, this effect can be expressed as:

2 $${\theta }_{i}={\mu }_{\Theta }+{u}_{i}$$

Consequently, the observed value can be expressed combining (1) and (2):

3 $${y}_{i}={\mu }_{\Theta }+{u}_{i}+{e}_{i}$$

Cochran (1954) proposed, under very general conditions, the following minimal variance estimate of *μ*_*Θ*_. It is a weighted mean, being the weights the inverse of the variance of the estimates that are averaged, ${w}_{i}=1/{\sigma }_{{y}_{i}}^{2}$(see formula 5):

4 $${\widehat{\mu }}_{\Theta }=\frac{\sum {w}_{i}\cdot {y}_{i}}{\sum {w}_{i}}$$

Thus, to obtain an estimate of the mean effect size with (4) it is necessary to have the variances of the studies (or their estimates). In the classic REM, (3), the *y* value is decomposed linearly into two independent sources of variation: the variability of the parametric effect size, and the sampling variability of *y* around *θ*. Each source of variation is represented as a continuous random variable (*crv*) with a certain probability density function (*pdf*). The *crv* that refers to the parametric value of the effect size has an expected value of 0 and variance *τ*^2^; the *crv* referring to the sampling of *e* has mean 0 and variance ${\sigma }_{e}^{2}$. It is also assumed that *θ* and *e* are two independent *crv* (this assumption holds for some ES indices but not for others, as we will show later). As a consequence, under the REM the variance of *y* has traditionally been expressed as:

5 $${\sigma }_{y}^{2}={\sigma }_{u}^{2}+{\sigma }_{e}^{2}={\tau }^{2}+{\sigma }_{e}^{2}$$

Apparently, the distribution of *y* should be obtained through the convolution of the *pdfs* of the two *crv* involved in (3). However, Eq. (5) is usually managed replacing ${\sigma }_{e}^{2}$ with ${\sigma }_{e/\theta }^{2}$. In fact, expression (5) is the formula (7) in Hedges (1983), where *τ*^2^ is the variance of the true effects, and ${\sigma }_{e}^{2}$ is the sampling variance of *e*, conditioned to a specific value of *θ*.

Before continuing we should make explicit a fundamental distinction in any formulation of a meta-analytical REM. On one hand, we have the variance of *y* at the *study level*. The variance of *y* in a specific study has to do with the uncertainty about *y* in that study. Since it is the variance of the distribution of *y* for that specific study, the uncertainty about *e* must take into account the full range of *θ*, and not only the conditional variance for the specific *u* in that study.

On the other hand, we have the variance of the distribution of *y* at the *meta-analysis level*. Remember that the meta-analyst is interested in modeling the distribution of *y* values obtained from a series of independent estimates, most probably obtained with different sample sizes. Each independent estimate has its own variance at the study level. However, as the variance of *y* in any study is also related with the uncertainty about *e* for the full range of *u*, the differences between the variances of the studies are entirely due to the different sample sizes of the studies, and not to the specific value of *δ* in the study.

Then, formula (5) is the variance of the distribution of *y* at the study level. The meta-analyst has a sample of independent values, *y*_1_, *y*_2_,…, *y*_k_, each with its own variance at the study level. The distribution of *y* at the meta-analysis level is the compound of *k* distributions at the study level, and cannot be expressed as in (5) if ${\sigma }_{e}^{2}$ is interpreted as ${\sigma }_{e/\theta }^{2}$. Unfortunately, on many occasions both interpretations, ${\sigma }_{e}^{2}$ and ${\sigma }_{e/\theta }^{2}$, are represented interchangeably with (5), without making explicit reference to which is which.

We are going to express the variance of *y* at the study level in a different way than (5), because this will allow us to highlight why for some ES indices (5) is often misinterpreted, as is usually done for *g*.

To simplify the expressions that follow, we temporally define ${v}_{i}={u}_{i}+{e}_{i}$, so that ${\mu }_{y}={\mu }_{\Theta }$ and ${\sigma }_{y}^{2}={\sigma }_{V}^{2}$. In Appendix S1 (supplemental material) it is shown that, by changing the variable *w* = *v* – *u*, we arrive at the variables *w* and *u* being *linearly independent*, and that ${\sigma }_{V}^{2}$ is equal to:

6 $${\sigma }_{V}^{2}={\tau }^{2}+\underset{-\infty }{\overset{\infty }{\int }}\underset{-\infty }{\overset{\infty }{\int }}{w}^{2}\cdot {f}_{U}(u;0,\tau )\cdot {f}_{E}(w;0,{\sigma }_{e})dudw$$

In Appendix S1 we show that when ${\sigma }_{e}^{2}$ does not depend on the parametric value of the effect size, the double integral in (6) is just ${\sigma }_{e}^{2}$, and we obtain that the variance of *y* is ${\tau }^{2}+{\sigma }_{e}^{2}$, which is the classical expression of the decomposition of the variance of *y* in formula (5). However, when ${\sigma }_{e}^{2}$ depends on the parametric value, ${\sigma }_{V}^{2}$ cannot be expressed as in (6); instead, ${\sigma }_{V}^{2}$ is (see Appendix S1):

7 $${\sigma }_{V}^{2}={\tau }^{2}+\underset{-\infty }{\overset{\infty }{\int }}\underset{-\infty }{\overset{\infty }{\int }}{w}^{2}\cdot {f}_{U}\left(u;0,\tau \right)\cdot {f}_{E}\left(w/u;0,{\sigma }_{e}\left(u,{\mu }_{\Theta },m,\widetilde{\text{n}}\right)\right)dudw$$

The variance of *v* is different when the variance depends on the parameter than when it does not depend on it. In Appendix S1 we apply (7) to the case of *g*. Specifically, as the conditional variance of *g* is a function of *δ*, the variance of *g* at the study level should not be written as in (5) or (6); instead it should be:

8 $$\begin{aligned}{\sigma }_{G}^{2}&={\tau }^{2}+E\left({\sigma }_{V}^{2}/\delta \right)\\ &={\tau }^{2}+\underset{-\infty }{\overset{\infty }{\int }}\underset{-\infty }{\overset{\infty }{\int }}{w}^{2}\cdot {f}_{U}\left(u;0,\tau \right)\cdot {f}_{E}(w/u;0,{\sigma }_{e}(u,{\mu }_{\Delta },m,\widetilde{\text{n}}))dudw\end{aligned}$$

In words, for all of the above, in general the REM cannot be applied interchangeably and with the same formulas with ES indices whose conditional variances depend on the value of the parameter, such as *g*, as with indices whose variances are stochastically independent of the parameter. When the variances are independent of the parameter, the variance of the distribution of the ES index at the study level is (5), while when the variances depend stochastically on the parameter, as in *g*, the general formula for the variance of the distribution is (7), while (8) expresses the same for the specific case of *g*.

## The practical problems of the random effects model with *g*

The practical work with the REM with *g* is usually done, questionably, by calculating the variance of *g* at the study level as the sum of the variance of the parametric effects, *τ*^2^, and the conditional variance of *e* given the specific value of *δ* in that study:

9 $$\begin{aligned}{\sigma }_{G}^{2}&={\tau }^{2}+\underset{-\infty }{\overset{\infty }{\int }}{e}^{2}{f}_{E}(e;\delta )de\\ &={\tau }^{2}+{\sigma }^{2}(e/\delta )\end{aligned}$$

As the *δ* value of a given study is unknown, the integral in (9) is usually replaced by formula (A5) in Appendix A, or one of the approximations available (Suero et al., 2023). However, (9) is not the correct formula for the variance of *g* at the study level. As explained above, the reason is that the uncertainty on *g* in any specific study comes from the *e* values for the whole range of *δ* values, and not only for any specific value of *δ*. The correct variance of *g* at the study level is (8), not (9). In other words, the decomposition of the sources of variation has not had an effect on the sampling variability (variance of *e*, given a value of *δ*), which continues to depend on the value of the parametric effect size. Probably in most common meta-analytic scenarios, the difference between (8) and (9) is small, and assuming independence between *δ* and the variance of *g* generates negligible errors. Still, the covariation exists and can have unexpected consequences in others unanticipated contexts. Of course, in ES indices in which the variance of *y* does not depend on the parameter, the conditional variance with a given pair of sample sizes is a constant and (9) is equivalent to (8).

Beyond that fundamental flaw, the way the variance of *g* has been traditionally managed in REM, (9), has created new problems. First, the weighted combination of the estimates, formula (4), is no longer the minimum variance estimate of *μ*_*Δ*_, since for it to be more precise, the weights would have to be independent of the combined values. The positive relationship between *g* and its conditional variance (negative relationship with its weight), as expressed in (A4), gives place to underestimates of *μ*_*Δ*_. However, in practical work the conditional variance is obtained through (A5) or any of the approximate formulas available (Suero et al., 2023). Formula (A5) shows that, added to the covariation between *δ* and the *true variance* conditioned on *δ*, there is another source of covariation between *g* and its *estimated variance* conditioned on $\widehat{\delta }$. That is, for a given *δ* and a fixed sample size, the estimated variance of *g* depends on the estimated value of *g*. This point and the consequent bias when estimating the mean effect weighting the estimates by the inverse of their variances has already been noted by Hedges in the early days of meta-analysis (Hedges, 1982, 1983; Hedges & Olkin, 1985). However, as we have argued earlier, this problematic covariation is introduced by the questionable practice of using the conditional variance (9) instead the unconditional one (8). As we will see, if the variance of the distribution (the unconditional one) is used, then there is no covariation between *g* and its variance at the study level.

Second, the use of conditional variances instead of unconditional ones can have consequences in any analysis in which they intervene. For example, that will occur in any estimator of *τ*^2^ that involves the studies’ variances, such as the Hedges estimator itself, sometimes known as Cochrane–Hedges ANOVA. The same is true in all cases in which the estimation involves a first step with a provisional estimate of *μ*_*Δ*_, which is obtained by weighting the inverses of the variances (for example, under the fixed effect model).

In addition to the previous problems, it is often also assumed that *δ* (or *μ*_*i*_) is normally distributed. Then, following (3) it is also assumed that *g* is normally distributed. But that is not true, because for any given value of *δ*, *g* is distributed as a transformed student’s *t* (Appendix A). Even though only with large samples does the distribution of *g* approach normality, in practical work normality is assumed regardless of the size of the samples. Assuming normality in *δ* does not imply normality in *g*, but this distributional restriction is not necessary to reach the *pdf* of the distribution of *g*, as we will see later.

An additional problem that has also been frequently highlighted is that in the classical REM model, the weights are taken as known, when in fact they are estimated. This is because the weights are equal to the inverses of the variances of the studies, which themselves are actually estimated. This problem is not solved with our alternative formulation of the REM, in which the variances are also estimated. However, we will see that the accuracy of the variances estimated with the MM (formula 8) is larger than with the classical REM (formula 9).

Now, let’s go back to the distribution of *g* at the meta-analysis level. The sample of observed *g* values have different variances in their distributions at the study level. However, all of them have in common the fact that their *δ* values come from a single distribution, with mean *μ*_*Δ*_ and variance *τ*^2^. Given that the *g* values are independent, the expected variance in a sample of *k* observations with specific sample sizes is:

10 $$E({S}_{g}^{2})={\tau }^{2}+\frac{1}{k}\sum_{i=1}^{k}E({\sigma }_{e}^{2})$$

Of course, the questionable practice of using the conditional variances instead of the unconditional variances can be reproduced if the conditionals, ${\sigma }_{e/\theta }^{2}$, are used in (10), instead of the unconditional, ${\sigma }_{e}^{2}$. However, the magnitude of the error is often considered negligible, small enough for the practical equations being useful in many real scenarios. In fact, Hedges (1983) proposed an estimate of *τ*^2^ based on (10) and on the estimated conditional variances that perform very well in a broad range of scenarios (e.g., Blázquez-Rincón et al., 2023):

11 $${\widehat{\tau }}^{2}={S}_{g}^{2}-\frac{1}{k}\sum_{i=1}^{k}{\widehat{\sigma }}_{e/{\delta }_{i}}^{2}$$

The fundamental flaw in the way the classic REM is usually applied to *g* comes from the fact that the conditional sampling variance of *e* depends on the own parameter, *δ*. It is possible to make a mistake taking the conditional instead of the unconditional variance only when they are different. That also happens, for example, in meta-analysis of Pearson’s correlation coefficients, for which the sampling variance is estimated with $(1-{r}^{2})/(N-2)$. However, in meta-analysis the *r* values are often replaced by their Fisher’s *Z* transformation, and the approximate variance of the transformed values, 1/(*N*-3), does not depend on the true correlation, *ρ*. The problem is circumvented with the transformation.

Many authors have highlighted the problems described here and sometimes have proposed solutions for some of them (see, for example Bakbergenuly et al., 2020; Böhning et al., 2002; Friedman, 2000; Hamman et al., 2018; Lin & Aloe, 2021). Most solutions are still handled within the framework of a components of variance logic. Then, the variances at the study level must be obtained for calculating the estimated mean effect, (4), and for deriving estimators of *τ*^2^, most often through the *Q* statistic. However, the solutions based on the *Q* statistic convey the well-known problems that challenge its use (e.g., Hedges, 2016; Hoaglin, 2016; Kulinskaya & Dollinger, 2016; Shuster, 2016).

Among the most interesting solutions is to replace the value of *g*_*i*_ in the formula of the variance of study *i* with the mean of *g* in the formulas of the variances of all studies. In this way the covariation between *g*_*i*_ and *w*_*i*_ vanishes. Another solution is to weight by some function of the study size (e.g., *ñ*). This solution also eliminates the covariation, and in addition the weights are known, not estimated. A recent simulation study (Buck et al., 2022) shows that in a realistic range of scenarios for psychology, employing various weighting schemes, including the uniform or unweighted one, produces small, often negligible, differences. However, we still want to weight by the inverse of the study variance for two reasons. The first is that we are looking for the optimal weights in terms of efficiency. The second is that, even if weighting with a different scheme for the pooled effect size estimate, we still need the study variances for other purposes. For example, we need the variance of the combined estimate, which is obtained through the variances of the studies, for inferential purposes, such as testing the null hypothesis of the mean effect or to obtain the confidence intervals around the combined estimate.

Alternatively, we describe in "Reformulating the REM for SMD as a mixture model" a formulation for the REM that does not incur most of the problems discussed above. Essentially, because the variances of *g* at the study level do not take part in the process of estimating *τ*^2^. Of course, when the variances are needed (for example, to weight the *g* values to estimate *μ*_*Δ*_), the unconditional instead of the conditional variances are used. The following formulation is a flexible alternative that encompasses both ES indices, in which the estimate and its estimated variance are stochastically dependent, and ES indices in which they are independent.

## Introduction to mixture models

What follows is a brief presentation of what a MM entails. For more detailed presentations, we refer to Böhning (2000), Everit (2013), Lindsay (1995), McLachlan et al. (2019), McLachlan and Peel (2000), Titterington et al. (1985).

In MM, a statistical population is defined as a mixture of two or more subpopulations. In this way, each of the elements of the population can belong to any of the mixed subpopulations. In formal terms, if a finite number *M* (which may be unknown) of subpopulations is assumed, the probability density function (*pdf*), *g*, of the statistical population, is defined as:

12 $$g(x;\boldsymbol{\Psi })=\sum_{i=1}^{M}{\pi }_{i}\cdot {f}_{i}(x;{{\varvec{\theta}}}_{i})$$

where *f*_*i*_ represents the *pdf* of each subpopulation or component; **θ**_***i***_ is the parameter vector of component *i*; the ${\pi }_{i}$ values represent the *mixing proportions*, which must satisfy the constraints that they take on non-negative values and that the sum of all of them is equal to 1; finally, **Ψ** represents a vector of parameters that includes the vectors **θ**_***i***_ and the vector of *mixing proportions*. The model defined in (12) is a *finite mixture model*, and the ordering $$(\begin{array}{ccc}{{\varvec{\theta}}}_{1}& ...& {{\varvec{\theta}}}_{M}\\ {\pi }_{1}& ...& {\pi }_{M}\end{array})$$ is the *mixing distribution*.

If there is an infinite number of subpopulations, then we define:

13 $$g(x;\boldsymbol{\Theta })=\int f(x;{\varvec{\theta}})\cdot h({\varvec{\theta}};\boldsymbol{\Theta })d{\varvec{\theta}}$$

where *h* is the *pdf* called the *mixing distribution* or *latent distribution*, and **Θ** is the vector of parameters of *h*. Note that in (13) it is assumed that all the components have the same distribution, but they could differ in their parameters. It can be considered that **θ** is a vector of random variables, so the expression inside the integral can be rewritten as:

14 $$g(x;\boldsymbol{\Theta })=\int f(x/{\varvec{\theta}})\cdot h({\varvec{\theta}};\boldsymbol{\Theta })d{\varvec{\theta}}$$

Thus, *g* is the distribution, with respect to **θ**, of a joint distribution of the random variables *x* and **θ**.

The great flexibility of this type of model allows it to be applied to a wide variety of statistical problems. Lindsay (1995) presents a list of different statistical models that can be interpreted as cases of MM, including random effects models. Treating them as MM allows relaxing some assumptions of the classic linear REM, such as the normality of parametric effects (see Jackson & White, 2018).

This MM offers a flexible structure that facilitates modeling when heterogeneity greater than that expected under a given statistical model is observed in a sample. Suppose, for example, that there is a sample of *k* values (*x*_*1*_,* x*_*2*_,…, *x*_*k*_) in which the observed heterogeneity is greater than expected with respect to a population. An explanatory hypothesis for this heterogeneity could be that the elements of the sample actually come from several subpopulations that define a population with greater heterogeneity than those of the subpopulations that compose it. Models such as those in (12) or (13) make it possible to define a population whose expected variability is greater than that of the subpopulations that define it. For example, MM have been applied to explain the presence of outliers (reflected as skewness) in the distribution of ES values (Beath, 2014). Specifically, the empirical distribution would be a mixture of two distributions, both described as in the REM; one generates the bulk of the values and the other generates the outliers. In this way, Beath’s (2014) proposal allows an alternative explanation for the skewness of the funnel plot, unrelated to publication bias. It could be that a small number of studies, with a common characteristic that defines a particular subpopulation, contribute ES values that are generally higher (or lower) from the rest. The low probability of this type of study can generate a distribution in which there are a few discordant values that generate the observed skewness.

## Reformulating the REM for SMD as a mixture model

Readers less interested in the technical aspects of the argument can skip this section without losing track of the main arguments. The information typically available for a meta-analysis with SMD is a set of effect size estimates (*g*), obtained from pairs of samples of *N*_1_ and *N*_2_ observations, contributed by the primary studies. The *g* values can be represented as a sample of values drawn from a population characterized by a given *pdf* and obtained through *N*_1_ and *N*_2_ observations. From that sample, the parameter or parameters of interest can be estimated. Let us assume for a moment that the parameter *δ* is constant in the studies (the so-called *fixed effect* model, also called *common effect* model). Then the *g* values of a meta-analysis constitute a sample drawn from a *pdf* whose variance is just the sampling variance, ${\sigma }_{e}^{2}$, and their expected value is *δ*. From this sample, we want to estimate *δ*.

In a more realistic case, where it is assumed that the parameter varies across the studies (REM), it is understood that *δ* is a continuous random variable. Therefore, each of the sample values of *g* can come from subpopulations with different unknown parameter values, $\delta$. Consequently, we have two continuous random variables, *g* and *δ*, with their joint *pdf*, $f(g,\delta )$.

Now, the only observed values are the *g*s and the samples sizes. We need to define the marginal distribution of *g*. The joint *pdf*, $f(g,\delta )$, can be expressed as:

15 $$f(g,\delta )={f}_{G}(g/\delta )\cdot {f}_{\Delta }(\delta )$$

where ${f}_{G}(g/\delta )$ is the *pdf* of *g* conditional on a value of *δ*, and *f*_Δ_ is the *pdf* of $\delta$ with mean *μ*_Δ_ and variance *τ*^2^. From (15), the marginal *pdf* of *g* is defined by the expression:

16 $${h}_{G}(g;{\mu }_{\Delta },\tau )=\underset{-\infty }{\overset{\infty }{\int }}{f}_{G}(g/\delta )\cdot {f}_{\Delta }(\delta ;{\mu }_{\Delta },\tau )d\delta$$

It is easy to see that (16) is nothing more than an application of (14), where *f*_Δ_ is the *mixing distribution* or *latent distribution*, and in the vector **Θ** of (14) are included the parameters *μ*_Δ_ and *τ*^2^.

In summary, the REM can be formulated as a MM (Böhning, 2000; Thomas, 1989). The *g* values are a sample drawn from *h*_*G*_ and the goal of the meta-analyst is to estimate the parameters *μ*_Δ_ and *τ*^2^ from that sample. To do this, we need to show the relationship between the parameters of *h*_*G*_ and the parameters *μ*_Δ_ and *τ*^2^ of the *mixing* or *latent distribution*. We do that in "Distribution of G for fixed sample sizes" and "Distribution of G for variable sample sizes".

### Distribution of G for fixed sample sizes

For the development of expressions and demonstrations, the nomenclature of Hedges (1981) will be followed (see the definitions of *m*, *c*(*m*) and *ñ* in Appendix A).

We have shown that the REM can be defined as a MM:

17 $${h}_{G}(g;{\mu }_{\Delta },\tau ,m,\widetilde{\text{n}})=\underset{-\infty }{\overset{\infty }{\int }}{f}_{G}(g/\delta ;m,\widetilde{\text{n}})\cdot {f}_{\Delta }(\delta ;{\mu }_{\Delta },\tau )d\delta$$

The centered moment of order *r* of *g* is defined as:

18 $${\text{E}}[{(g-{\mu }_{G})}^{r}]=\underset{-\infty }{\overset{\infty }{\int }}{(g-{\mu }_{G})}^{r}\cdot {h}_{G}(g;{\mu }_{\Delta },\tau ,m,\widetilde{\text{n}})dg$$

In Appendix S2 (see S2-10 to S2-12, supplemental material) we show that:

19 $${{\mu }_{G}=\mu }_{\Delta }.$$

And a general expression has been derived that obtains the centered moment of order *r* (S2-1 to S2-13):

20 $${\text{E}}\left[{\left(g-{\mu }_{\Delta }\right)}^{r}\right]=\sum_{k=0}^{r}{\left(-1\right)}^{k}\bullet \frac{r!}{k!\cdot \left(r-k\right)!}\bullet {\left(c\left(m\right)\cdot \frac{1}{\sqrt{\widetilde{\text{n}}}}\right)}^{r-k}\bullet {\mu }_{\Delta }^{k}\bullet {\left(\frac{1}{2}\cdot m\right)}^{r/2}\cdot \frac{\Gamma \left(\frac{1}{2}\cdot \left(m-\left(r-k\right)\right)\right)}{\Gamma \left(\frac{1}{2}\cdot m\right)}\cdot \sum_{j=0}^{\frac{r-k}{2}}\left(\begin{array}{c}r-k\\ 2\cdot j\end{array}\right)\cdot \frac{\left(2\cdot j\right)!}{{2}^{j}\cdot j!}\cdot {\left(\sqrt{\widetilde{\text{n}}}\right)}^{\left(r-k\right)-2\cdot j}\cdot {\text{E}}\left({\delta }^{\left(r-k\right)-2\cdot j}\right)$$

Applying (20) for the second-order moment we conclude that the variance of *G* is equal to (formula 21b is the same, expressed in terms of the simplified Hedges’ nomenclature of coefficient *a*):

21a $${\sigma }_{G}^{2}=\frac{c{(m)}^{2}\cdot m}{(m-2)\cdot \tilde{n} }\cdot (1+\tilde{n} \cdot ({\tau }^{2}+{\mu }_{\Delta }^{2}))-{\mu }_{\Delta }^{2}$$

21b $${\sigma }_{G}^{2}=\frac{a}{\tilde{n} }[1+\tilde{n} \cdot ({\tau }^{2}+{\mu }_{\Delta }^{2})]-{\mu }_{\Delta }^{2}$$

Applying again (20), the third-order moment is:

22 $${\text{E}}_{{f}_{G}}[{(g-{\mu }_{\Delta })}^{3}]=\frac{c{(m)}^{3}}{\tilde{n} }\cdot \frac{{m}^{3/2}\cdot \Gamma (\frac{m-3}{2})}{2\cdot \sqrt{2}\cdot \Gamma (\frac{m}{2})}\cdot (3\cdot {\mu }_{\Delta }+{\text{E}}_{{f}_{\Delta }}[{\delta }^{3}]\cdot \tilde{n} )-3\cdot {\mu }_{\Delta }\cdot {\sigma }_{G}^{2}-{\mu }_{\Delta }^{3}$$

So the skewness is:

23 $$\gamma =\frac{E[{(g-{\mu }_{\Delta })}^{3}]}{{\sigma }_{G}^{3}}$$

The formula of the skewness, (23), allows us to study the consequences of the skewness of the distribution of *δ*. For example, even if the distribution of *δ* is symmetric (e.g., normal) the distribution of *G is* *skewed*. This characteristic contradicts the common assumption that the marginal distribution of *g* is normal (or symmetric).

Compared with the classic REM, it is important to note that to obtain the expressions (19), (21), and (23) it is only assumed that the individual scores of the two populations follow homoscedastic normal distributions (Appendix A). It is not necessary to assume any distribution for the *crv* Δ, since all three expressions hold regardless of the distribution of *δ*. Furthermore, it should be noted that ${\sigma }_{G}^{2}$ is not defined, as in the REM, by means of the (conditional) sampling variances of the values of the estimator of *g*, nor as the sum of two variances ${\tau }^{2}+{\sigma }_{e}^{2}$. The sampling variances, ${\sigma }_{e}^{2}$, do not appear in the formulas, which has the important consequence that it is not necessary to assume independence between the parameter value and the sampling variance (in fact, their potential dependence is implicitly assumed in the formulas).

In short, the model for fixed sample sizes is an infinite MM. An empirical distribution of *k* values of *g* would come in this case from a mixture of *k* of the infinite possible distributions, each of which provides only one empirical realization. However, they share the source of their parameter values (*δ*): a latent distribution with mean *μ*_Δ_ and variance *τ*^2^. *The* *MM for a fixed pair of sample sizes is the distribution of g at the study level.*

### Distribution of G for variable sample sizes

In formulas (21) and (22), unknown but constant quantities appear, such as *τ*^2^ and *μ*_*Δ*_, but also known quantities appear, such as the sample sizes *(N*_*1*_ and *N*_*2*_), implicit in *m* and *ñ*. Given the typical ranges of values of *g* and sample sizes in many fields of psychology, it is clear from formula (A4) that the magnitude of the variance of *g* depends above all on the known quantities. Importantly, the known quantities (*N*_*1*_ and *N*_*2*_) do not represent random variables but rather arbitrarily fixed, known magnitudes, which are part of the studies’ designs. Obviously, the sample sizes of the *k* studies will generally be different from study to study, so the variances at the study level will be different due to the variations of their sample sizes.

Suppose *k* primary studies, each of which has associated sample sizes *N*_*i1*_ and *N*_*i2*_; those values are implicit in *m*_*i*_ and *ñ*_*i*_ (*I* = 1, 2, …, *k*). The *g* value of each of the primary studies is a value from a statistical population that follows the *pdf* ${h}_{G}(g;{\mu }_{\Delta },\tau ,{m}_{i},{\tilde{n} }_{i})$. Note that, with respect to the *pdf*, the different primary studies only differ in *m*_*i*_ and *ñ*_*i*_, the rest of the parameters being the same (${\mu }_{\Delta }$ and $\tau$). Accordingly, the *pdf* of the *g* values from the *k* primary studies is:

24 $${g}_{G}(g;{\mu }_{\Delta },\tau ,{\varvec{m}},\boldsymbol{\tilde{n} })=\sum_{i=1}^{k}\frac{1}{k}\cdot {h}_{G}(g;{\mu }_{\Delta },\tau ,{m}_{i},{\tilde{n} }_{i})$$

where **m** and **ñ** are vectors with elements *m*_*i*_ and *ñ*_*i*_, respectively (*i* = 1, 2, …, *k*).

It is important to reiterate that the sample sizes in a study, *N*_1*i*_ and *N*_2*i*_, should not be considered as random components. Nor do the sample sizes of the studies in a meta-analysis belong to a supposed population of sample sizes they represent. Sample sizes are known, arbitrarily set quantities whose magnitude is unrelated to the random variable that reflects the effect under study, as expressed in formula (1). The variations that are of interest to the meta-analyst are the values of *δ*, which are reflected in the two unknown parameters, *μ*_*Δ*_ and *τ*^2^. In order to estimate these parameters, it is necessary to derive formulas that reflect the variety in the sample sizes but take those quantities as known magnitudes.

Equation (24) can be interpreted as a *finite mixture distribution* with *k* components, each with its own *pdf*, and 1/*k* represents the *mixing proportion* for all the components. Note that the *mixing proportions* are the same for all components; they could be modified if it were taken into account that there may be primary studies that share the values of *m* and *ñ* (the same sample sizes), but it is not necessary.

What distinguishes the *finite mixture distribution* defined in (24) from other applications of mixture models is that several things are known: (a) the number of components, which is equal to the number of studies, *k*; and (b) given the value of *g* of a primary study, it is known to which component of the *finite mixture* it belongs, since the samples sizes are known.

In short, we have a *finite mixture distribution* with *k* components (the proportion of *g* values coming from each component is 1/*k*). Nevertheless, as we described in the previous section, each component of this finite mixture distribution is in turn an *infinite mixture distribution* in which the infinite values of its components are defined by the range of *δ*. Importantly, only one observed value is available from each of the *k* infinite mixture distributions.

Taking into account what has been said up to now, we want to derive expressions for the mean, the variance, and the skewness of ${g}_{G}$ in the scenario involving different sample sizes. For this, the *law of total expectation*, the *law of total variance* and the *law of total* *cumulance* will be applied.

To obtain the expected value, we apply the *law of total expectation*:

25 $${\text{E}}(G)=\frac{1}{k}\cdot \sum_{i=1}^{k}{\text{E}}(G/{m}_{i},{\tilde{n} }_{i})$$

As we have established in (19) that ${\text{E}}(G)={\mu }_{\Delta }$, the expected value does not depend on *m*_*i*_ or *ñ*_*i*_, therefore:

26 $${\text{E}}(G)=\frac{1}{k}\cdot \sum_{i=1}^{k}{\mu }_{\Delta }$$

27 $${\mu }_{G}={\mu }_{\Delta }$$

To obtain the variance we apply the *law of total variance*:

28 $${\text{Var}}(G)={\text{E}}[{\text{Var}}(G/{m}_{i},{\tilde{n} }_{i})]+{\text{Var}}[{\text{E}}(G/{m}_{i},{\tilde{n} }_{i})]$$

Now, given that ${\text{E}}(G/{m}_{i},{\tilde{n} }_{i})={\mu }_{\Delta }$, then the variance of the conditional expected values is equal to zero; so:

29 $${\text{Var}}(G)={\text{E}}[{\text{Var}}(G/{m}_{i},{\tilde{n} }_{i})]$$

In (21), we found the formula for the variance of *g* for specific values of *m* and *ñ*; therefore:

30 $$\text{Var}(G)=\frac1k\cdot\sum_{i=1}^k\left[\frac{c{(m_i)}^2\cdot m_i}{(m_i-2)\cdot{\widetilde n}_i}\cdot(1+{\widetilde n}_i\cdot(\tau^2+\mu_\Delta^2))-\mu_\Delta^2\right]$$

Simplifying, we arrive at:

31 $${\sigma }_{G}^{2}=\frac{1}{k}\cdot \sum_{i=1}^{k}[\frac{c{({m}_{i})}^{2}\cdot {m}_{i}}{({m}_{i}-2)\cdot {\tilde{n} }_{i}}\cdot (1+{\tilde{n} }_{i}\cdot ({\tau }^{2}+{\mu }_{\Delta }^{2}))]-{\mu }_{\Delta }^{2}$$

An alternative way of expressing this formula, with the nomenclature of Hedges (1983; see Appendix A), is the following:

32 $${\sigma }_{G}^{2}=\frac{1}{k}\sum_{i=1}^{k}\frac{{a}_{i}}{{\tilde{n} }_{i}}[1+{\tilde{n} }_{i}\cdot ({\tau }^{2}+{\mu }_{\Delta }^{2})]-{\mu }_{\Delta }^{2}$$

In the end, the variance of *g* at the meta-analysis level is the mean of the *k* variances of *g* at the study level (${\sigma }_{{G}_{i}}^{2}$). These variances share the values of the parameters, while they differ in their *a*_*i*_ and *ñ*_*i*_, which exclusively reflect the sample sizes. Importantly, the *k* variances of *g* are not specifically related to the *g* value of their study. There is no dependency between *g*_*i*_ and ${\sigma }_{{G}_{i}}^{2}$. In order to better reflect this fact, a more strict terminology can be used to represent the variance as ${\sigma }_{G}^{2}({a}_{i},{\tilde{n} }_{i})=(\frac{{a}_{i}}{{\tilde{n} }_{i}}+{a}_{i}\cdot ({\tau }^{2}+{\mu }_{\Delta }^{2}))-{\mu }_{\Delta }^{2}$. However, we simplify it by representing the variance of *g* at the study level as ${\sigma }_{{G}_{i}}^{2}$. The differences between the variances of the studies are only in the sample sizes (as reflected in *a*_*i*_ and *ñ*_*i*_).

To obtain the skewness, we can apply the *law of total* *cumulance* for the third-order moment:

33 $${\mu }_{3}(G)={\text{E}}[{\mu }_{3}(G/{m}_{i},{\tilde{n} }_{i})]+{\mu }_{3}[{\text{E}}(G/{m}_{i},{\tilde{n} }_{i})]+3\cdot {\text{Cov}}[{\text{E}}(G/{m}_{i},{\tilde{n} }_{i}),{\text{Var}}(G/{m}_{i},{\tilde{n} }_{i})]$$

where ${\mu }_{3}$ denotes the centered moment of third-order.

As in the case of the total variance (28), ${\text{E}}(G/{m}_{i},{\tilde{n} }_{i})={\mu }_{\Delta }$; that is, the expected value does not depend on the sample size. Then, the second and the third addends in the previous equation are equal to zero, and actually Eq. (33) is:

34 $${\mu }_{3}(G)={\text{E}}[{\mu }_{3}(G/{m}_{i},{\tilde{n} }_{i})]$$

We know, (22), the formula for the third-order moment of *g* for a given value of *m*, therefore:

35 $${\mu }_{3}(G)=\sum_{i=1}^{k}\frac{1}{k}\cdot [\frac{c{({m}_{i})}^{3}}{{\tilde{n} }_{i}}\cdot \frac{{m}_{i}^{3/2}\cdot \Gamma (\frac{{m}_{i}-3}{2})}{2\cdot \sqrt{2}\cdot \Gamma (\frac{{m}_{i}}{2})}\cdot (3\cdot {\mu }_{\Delta }+{\text{E}}_{{f}_{\Delta }}[{\delta }^{3}]\cdot {\tilde{n} }_{i})-3\cdot {\mu }_{\Delta }\cdot {\text{Var}}(G/{m}_{i})-{\mu }_{\Delta }^{3}]$$

The skewness is:

36 $$\gamma =\frac{{\mu }_{3}(G)}{{\sigma }_{G}^{3}}$$

where the denominator is the cubed total standard deviation; that is, the square root of (31):

37 $${\sigma }_{G}^{3}={[\sqrt{\sum_{i=1}^{K}\frac{1}{K}\cdot [\frac{{a}_{i}}{{\tilde{n} }_{i}}\cdot (1+{\tilde{n} }_{i}\cdot ({\tau }^{2}+{\mu }_{\Delta }^{2}))]-{\mu }_{\Delta }^{2}}]}^{3}$$

Thus, the model for varied sample sizes is a finite MM of infinite MMs. Specifically, the empirical distribution comes from a finite mixture of *k* distributions or components, each defined by a pair of sample sizes (*N*_1_, *N*_2_). However, each of those components is in turn an infinite mixture distribution that provides only one empirical realization. As in the fixed sample-size model, the components share the source of their parameter values (*δ*): a latent distribution with mean *μ*_Δ_ and variance *τ*^2^. *The* *MM of g at the meta-analysis level, that is for varied sample sizes, is a finite MM composed by k infinite MMs*.

Recently, Bakbergenuly et al. (2022) have developed a general formulation that leads to expressions that can be applied to any ES whose distribution is known. Here, we have focused on the SMD, and offer the specific formulas for *g*. However, we believe that using conditional or unconditional variances should not respond to preferences. Only the unconditional ones are conceptually correct, and other problems are avoided.

## Checking the accuracy of the formulas

In this section, we empirically check the accuracy of the formulas for the mean, variance, and skewness, both for a case of fixed sample size and for a case of variable sample sizes. We do not need an exhaustive simulation study; rather, we want only to show an exemplar case of the predictive capacity of these formulas. The *R* code is in Appendix S3 (supplemental material). We have extracted scores of individuals for 10 million primary studies where the final result is a *g* value. The statistics of that distribution of *g* values are compared with those predicted by the MM.

For each primary study, two sets of individual scores were extracted, ***X***_*1*_ and ***X***_*2*_, corresponding to the two populations of sizes *N*_*1*_ = *N*_*2*_ = 15 for the case of fixed sample sizes. For the case of variable samples sizes, there were 8 different sizes [(5;5), (10;10), (5;15), (15;15), (10;20), (20;20), (10;30), (15;25)], which were extracted for the primary studies with uniform probabilities (1/8). The *δ* value for each primary study was extracted from a normal distribution with ${\mu }_{\Delta }=0.5$ and ${\tau }^{2}=0.1$. The scores of the two populations were drawn from the distributions N(0, 1) and N(*δ*, 1), respectively. The value of *g* was obtained through the conventional formulas (Appendix A).

In both the fixed sizes and variable sizes conditions, we have obtained the empirical mean, variance, and skewness index, as well as the values given by formulas (19), (21) and (23) for fixed sizes, and (27), (31) and (36) for variable sizes. The results, shown in Table 1, reflect an excellent fit between the empirical values and those provided by the formulas. The discrepancies are at least in the fourth decimal place in all cases. The empirical results are consistent with the predictions made from the proposed MM. Especially important is the accuracy of the variance obtained with (8) instead (9).

**Table 1:** Empirical and theoretical mean, variance, and skewness, as well as the discrepancy between them, with fixed sizes of *N*_1_ = *N*_2_ = 15 and variable sizes (see text)

	Fixed sample sizes			Variable sample sizes		
Statistic/parameter	Empirical	Theoretical	Theoretical—empirical	Empirical	Theoretical	Theoretical—empirical
Mean	0.49989	0.50000	0.00011	0.50012	0.50000	– 0.00012
variance	0.24263	0.24272	0.00009	0.30356	0.30380	0.00024
skewness	0.12047	0.12139	0.00092	0.22241	0.22161	– 0.00080

**Fig. 1:**
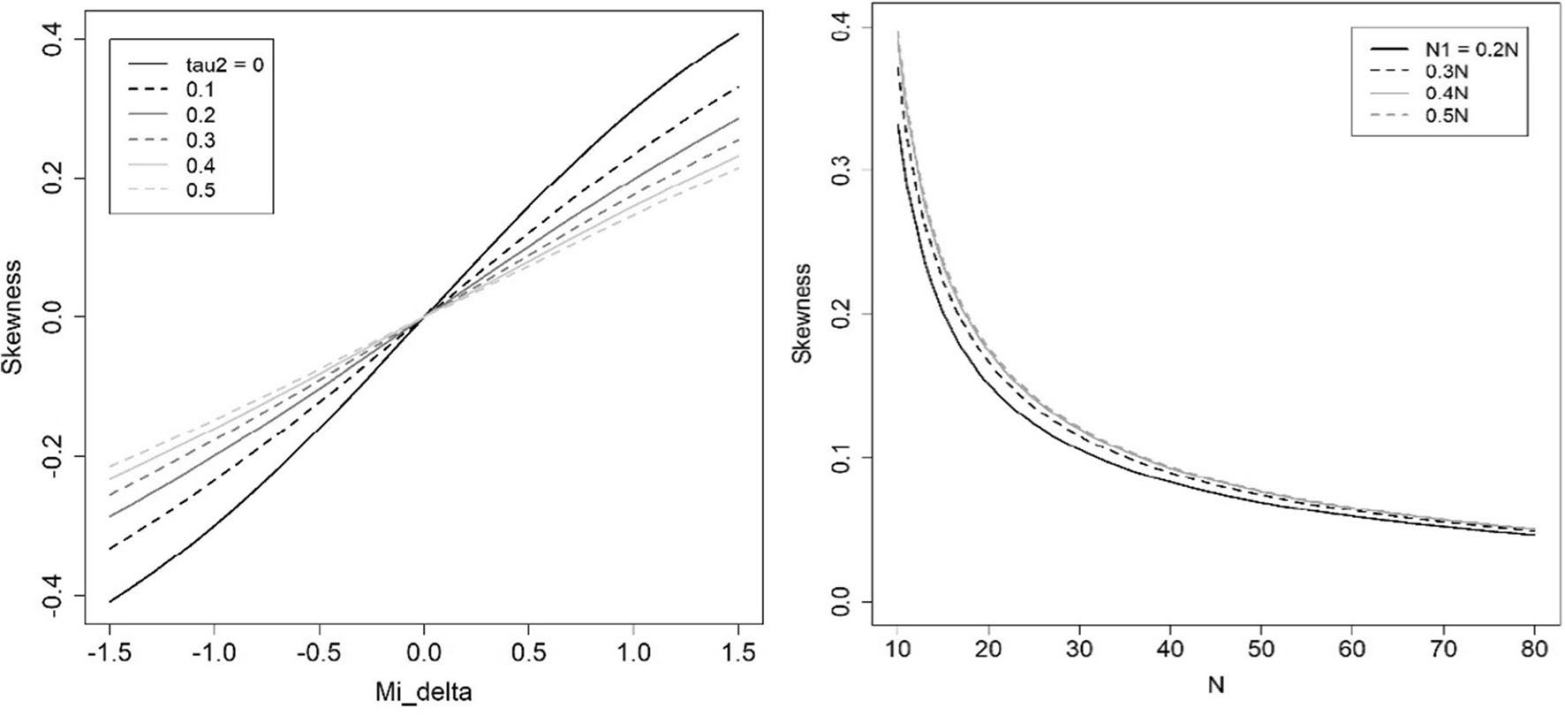
Degree of skewness of the distribution of *g* as a function of: **a** left, the parameters (*μ*_*δ*_ and *τ*^2^), for a case of fixed sample sizes (*N*_1_ = *N*_2_ = 15); **b** right, the total sample size and the imbalance between *N*_1_ and *N*_2_, for the values of the parameters used in the simulation (*μ*_*δ*_ = 0.50; *τ*.^2^ = 0.10)

With respect to skewness, the main conclusion is, as expected, that neither the distribution of *g* at the study level nor at the MA level is symmetrical. Furthermore, as can be seen in the formulas, the degree of skewness of the distribution of *g* depends both on the parameters, *μ*_*Δ*_ and *τ*^2^, and on the sample sizes. Some results obtained with these formulas will help the reader to get an idea of the degree of skewness of the distribution of *g* in a range of conditions that include most meta-analysis with *g* in psychology. Figure 1 (left) shows the degree of skewness calculated with (23), depending on the parameters (*μ*_*Δ*_ and *τ*^2^), for the same sample sizes used for fixed samples in the simulation (*N*_1_ = *N*_2_ = 15). The skewness is greater the larger the mean effect size (*μ*_*δ*_), but at the same time, it is greater the smaller the specific variance (*τ*^2^). Figure 1 (right) shows the degree of skewness, calculated with (36), depending on the sample sizes, for the values of the parameters used in the simulation. We have calculated the skewness for different imbalances between the sizes (e.g., for a total *N* of 20, the size of the first sample can be 0.2·*N* = 4, 0.3·*N* = 6, 0.4·*N* = 8 or 0.5·*N* = 10). Of course, the skewness is greater the smaller the total sample size. At the same time, however, the skewness is somewhat greater the greater the imbalance between *N*_1_ and *N*_2_, although the differences are rather small.

One of the main conclusions of the present research is that the skewness of the distribution of *g* will be somewhere between 0.0 and 0.30, approximately, with a typical value around 0.15. Statistical techniques in which the distribution of *g* is assumed to be symmetrical (typically normal) must be corrected to incorporate this feature. The MM is able to manage this feature, as formulas in previous sections allow for the prediction of skewness.

## Estimating the specific variance by the method of the moments

The first practical application of the described model should be a method to estimate the parameters of interest, *μ*_*Δ*_ and* τ*^2^. The easiest way to do this is to apply the method of moments. As $\overline{g }$ is an unbiased estimator of the mean effect size, (27) can be used to estimate *μ*_*Δ*_: ${\widehat{\mu }}_{\Delta }=\overline{g }$. However, the variance of ${\widehat{\mu }}_{\Delta }$ can be reduced if the estimates of the studies are weighted when combining the *g* values. Of course, we don’t want a weighting scheme that generates a dependency between *g* and its variance. As the variances of *g* at the study level obtained under the MM model are independent of the *g* value of the specific study, our weights can be the inverse of those variances (as suggested by Cochran) without generating an artificial dependency.

The proposed procedure involves three steps: (a) obtaining the estimation of *τ*^2^ with formula (39) (see below); (b) obtaining estimates of the variances of *g* at the study level with formula (21); and (c) obtaining the estimation of *μ*_*Δ*_, weighting the *g* values by the inverse of the variances obtained in the previous step.

For the first step, we can take formula (32) as the starting point; then:

38 $${\tau }^{2}=\frac{k\cdot [{\sigma }_{G}^{2}+{\mu }_{\Delta }^{2}]-\sum_{i=1}^{k}(\frac{{a}_{i}}{{\tilde{n} }_{i}})}{\sum_{i=1}^{k}{a}_{i}}-{\mu }_{\Delta }^{2}$$

After some tedious algebra (appendix B), we arrive at the following unbiased estimator of *τ*^2^:

39 $$\widehat\tau^2=\frac{\sum_{i=1}^k\;g_i^2-\sum_{i=1}^k\;\left({\displaystyle\frac{a_i}{{\widetilde n}_i}}\right)}{\sum\limits_{i=1}^ka_i}-\frac{\sum_{i=1}^k\;\frac{a_i}k}{\displaystyle\left(k-1\right)}\cdot\left[\frac{k\cdot\overline g^2-\sum_{i=1}^k{\displaystyle\frac{a_i}{k\cdot{\widetilde n}_i}}}{\sum_{i=1}^k\frac{a_i}k}-\frac{{\sum\limits_{i=1}^k}g_i^2-\sum_{i=1}^k\;\left(\frac{a_i}{{\widetilde n}_i}\right)}{\sum_{i=1}^ka_i}\right]$$

Formula (39) is very close, but not exactly the same, as the one reached by Malzahn et al. (2000).

In the second step, the variances at the study level are obtained through formula (21). In appendix C is shown how to obtain an unbiased estimate of the variance at the study level, ${\widehat{\sigma }}_{{G}_{i}}^{2}$. In practical terms, the expression ($\tau^2+\mu_\triangle^2$) in (21) is replaced with (C3):

$$\frac{\sum_{i=1}^k\;g_i^2-\sum_{i=1}^k\;\left({\displaystyle\frac{a_i}{{\widetilde n}_i}}\right)}{{\sum\limits_{i=1}^k}a_i}$$

Moreover, $\mu_{\mathit\Delta}^{2}$ is replaced with (C5):

$$\widehat\mu_\Delta^2=\frac{\sum\limits_{i=1}^k\frac{a_i}k}{\left(k-1\right)}\cdot\left[\frac{k\cdot\overline g^2-\sum_{i=1}^k\frac{a_i}{k\cdot{\widetilde n}_i}}{\sum_{i=1}^k\frac{a_i}k}-\frac{\sum_{i=1}^kg_i^2-{\sum\limits_{i=1}^k}\left(\frac{a_i}{{\widetilde n}_i}\right)}{\sum_i^k\;a_i}\right]$$

Once the variances of the studies, ${\widehat{\sigma }}_{{G}_{i}}^{2}$, are estimated in the third step, an unbiased estimate of *μ*_*Δ*_ is obtained weighting the *g* values by ${w}_{i}={~}^{1}\!\left/ \!{~}_{{\widehat{\sigma }}_{{G}_{i}}^{2}}\right.$:

40 $${\widehat\mu}_\Delta=\frac{\sum w_i\cdot g_i}{\sum w_i}$$

The estimated variance of the estimated mean effect is:

41 $${\widehat{\sigma }}_{{\widehat{\mu }}_{\Delta }}^{2}=\frac{1}{\sum {\widehat{w}}_{i}}=\frac{1}{\sum 1/{\widehat{\sigma }}_{{G}_{i}}^{2}}$$

Even at the risk of being redundant, it is important to note that the variances of *g* at the study level do not correlate with the *g* values themselves. In formulas (39) and (40), only the number of studies appears, the arithmetic mean of the *g* values ($\overline{g }$) and the sum of the *g*^2^ values (along with the sample sizes involved in *a* and *ñ*). The only difference between the variances is their sample sizes (as reflected in *a*_*i*_ and *ñ*_*i*_), but those are known amounts and do not reflect anything related to the focus of interest for the meta-analyst (the distribution of *δ*). As explained above, they are simply known amounts linked to the specific designs and should not be taken as random variables.

Other procedures have been proposed to avoid the covariation between the *g* values and their variances, including the use of the mean *g* in the formula of the conditional variances for all studies, the use of the known part of the formula of the variance (essentially the samples sizes, directly or as an effective samples sizes), and the use of the magnitude *a*/*ñ* as weights, etc. (see, for example,Hedges, 1982; Kulinskaya et al., 2021; Lin & Aloe, 2021). However, those alternatives are suboptimal, since, strictly speaking, they are not the variances of *g* at the study level. In that sense, weighting with the inverse of formula (21) should behave a little better as long as that is the formula for the variance of *g* of each study and it does not depend on the *g* or *δ* values of the own study.

In "The practical problems of the random effects model with g", we highlighted that another simplification of the classical REM model has also been frequently noticed, which consists of taking the variances of the studies as known rather than estimated. In the estimation procedure under the MM, the uncertainty that entails from the fact that the variances of the studies are estimated is also ignored. However, we believe that this problem is of much smaller magnitude in the MM model. The reason is that in the classic model the uncertainty of each variance is associated with that of the *g* value of that study. On the contrary, in the MM, all variances have their uncertainty associated with only two values, common to all studies, which are the estimates of *μ*_*Δ*_ and *τ*^2^. The variances of those two estimates are much smaller than the variances of the unknowns in the classical REM (the *g* values).

### A Monte Carlo simulation

In order to assess the performance of the estimators (39) and (40), we have run a Monte Carlo simulation with a handful of representative conditions. The conditions are the result of crossing the following factors: (a) the variance of true effects, *τ*^2^, with the values 0.10, 0.30, 0.50, 0.70, and 0.90; (b) the number of studies, *k*, with the values 15, 30, and 45. The sample sizes were taken from the same eight equiprobable pairs as in the simulation of Checking the accuracy of the formulas: (5;5), (10;10), (5;15), (15;15), (10;20), (20;20), (10;30) and (15;25). The mean effect size, ${\mu }_{\Delta }$, was fixed to 0.50. The number of replications is 20,000 (see Appendix S4, in the supplemental material, for the *R* code).

**Fig. 2:**
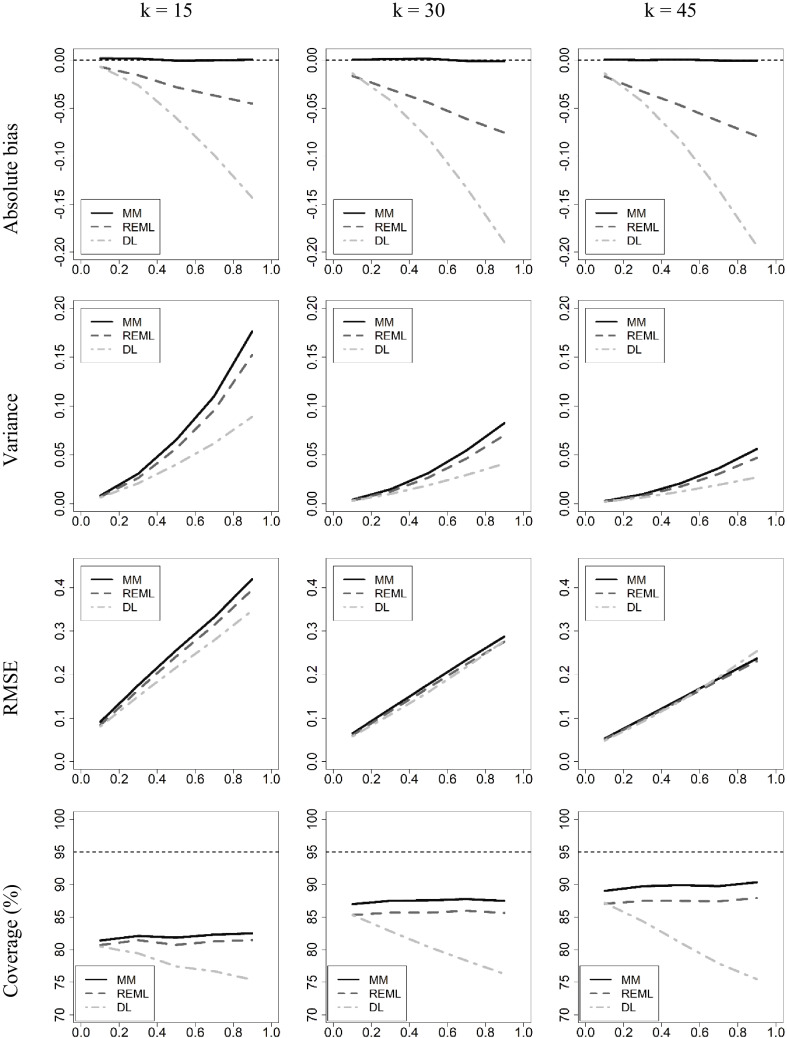
Performance of the estimator $\widehat\tau_{MM}^2$ as compared to both ${\widehat{\tau }}_{DL}^{2}$ and ${\widehat{\tau }}_{REML}^{2}$. In abscissa, the true *τ*^2^. The functions reflect the three estimators. In the columns, the three selected values of *k*

We have calculated, for each replica of each condition, the estimates of the specific variance by three methods: ${\widehat{\tau }}_{MM}^{2}$, ${\widehat{\tau }}_{DL}^{2}$ and ${\widehat{\tau }}_{REML}^{2}$ (the last two through metafor, Viechtabuer, 2010). We compare our estimator with the *DL* method (DerSimonian & Laird, 1986) because it is historically the more often used, and with the REM*L* method (Viechtbauer, 2005) because it is the most recommended, but there are many more estimators (Blazquez-Rincón et al., 2023; Petropoulou & Mavridis, 2017). The bias for each method is estimated through the average across estimates of the difference between the estimate and the generating parameter. The efficiency is estimated through the variance of the estimates in each condition. We first performed the simulation with no truncation of the negative variances, in order to have a better picture of their performance. Of course, the meta-analyst cannot accept and report a negative value of *τ*^2^, and must truncate negative estimates to zero, but this artificial manipulation could blur the properties of the estimators. However, as the differences with the truncated version were minimal and not systematic, we report only the results after truncation. Figure 2 shows the results for the estimates of *τ*^2^. In the first row are the results for the estimated bias; in the middle row are the variances of the estimates for each condition; in the lower row appear the *RMSE*. The abscissa shows the values of *τ*^2^, whereas in each column are the results for each of the three *k* values selected. The results reflect the lack of bias of $\widehat\tau_{MM}^2$, whereas ${\widehat{\tau }}_{REML}^{2}$ shows clearly less bias than ${\widehat{\tau }}_{DL}^{2}$. However, ${\widehat{\tau }}_{DL}^{2}$ is more efficient than the other two with the smaller *k*.

A poor estimator may appear to have good properties if its variance is artificially small. However, an artificially small variance ends up being detected in a too small coverage, because the confidence interval is too narrow. We believe that the lack of bias of ${\widehat{\tau }}_{MM}^{2}$ makes it preferable, since it is not compensated by the lower variance of the alternatives, especially ${\widehat{\tau }}_{DL}^{2}$. The best way to support this claim is to show that its confidence interval is closer to the nominal error rate. Unfortunately, we do not know the shape of the distribution of the three compared estimators. For this reason, we have chosen a method, nonparametric bootstrap, which, although in simulation studies usually appears as conservative in obtaining confidence intervals (e.g., Brannick et al., 2019; Veroniki et al., 2016; Viechtbauer, 2007) at least no risky assumptions are made. The results of those previous simulation studies indicate that the bootstrap CIs coverage is systematically lower than the nominal confidence level, since it provides narrower intervals than other procedures. In order to make a fair comparison between the chosen estimators, nonparametric bootstrap CIs were obtained for the three methods. Our reasoning is that if the nonparametric bootstrap method provides coverages closer to nominal level for ${\widehat{\tau }}_{MM}^{2}$ than ${\widehat{\tau }}_{REML}^{2}$ and ${\widehat{\tau }}_{DL}^{2}$, the coverage will also be greater and closer to the nominal level when the actual distribution of ${\widehat{\tau }}_{MM}^{2}$ is used to construct its CI. However, the latter remains pending for future developments.

Our procedure is as follows. Within each replica of the previous simulation, a bootstrap estimation process was opened, which consisted of obtaining 10,000 samples of the same size by simple resampling from the set of studies of the replica, with replacement. In each sample, the three estimators were obtained, in the same way as with the original sample of the replica. That is, ${\widehat{\tau }}_{MM}^{2}$ with our formulas above, and ${\widehat{\tau }}_{REML}^{2}$ and ${\widehat{\tau }}_{DL}^{2}$ with *metafor*. After the bootstrap process was completed, the 95% CI was defined by the 2.5th and 97.5th percentiles of the distribution of 10,000 estimates. Once the process was completed with the 20,000 replicates of each condition, the percentage of intervals that include the generating parametric value (*τ*^2^) was computed.

The results are shown in the last row of Fig. 2. As expected, the nonparametric bootstrap method generated intervals that were too conservative. With the three estimators and in all the conditions evaluated, the coverage remained below the nominal 95%. That said, it is also clear that the coverage intervals with ${\widehat{\tau }}_{MM}^{2}$ are closer to the nominal rate than those of the other two methods. With the three methods, coverage improves when going from 15 studies to 30 and then to 45. The *DL* method shows a significant worsening of coverage as *τ*^2^ increases. This does not happen with ${\widehat{\tau }}_{MM}^{2}$ and ${\widehat{\tau }}_{REML}^{2}$. However, the advantage of ${\widehat{\tau }}_{MM}^{2}$ over ${\widehat{\tau }}_{REML}^{2}$ increases with *k*. As long as we do not know the shape of the distribution of the proposed estimator, we will not be able to obtain more accurate confidence intervals. For now, we have not been able to reach this distribution, but the bootstrap results are promising and support our preference for ${\widehat{\tau }}_{MM}^{2}$ as an estimator of *τ*^2^, based essentially in the (lack of) bias and the (better) coverage of the confidence intervals.

**Fig. 3:**
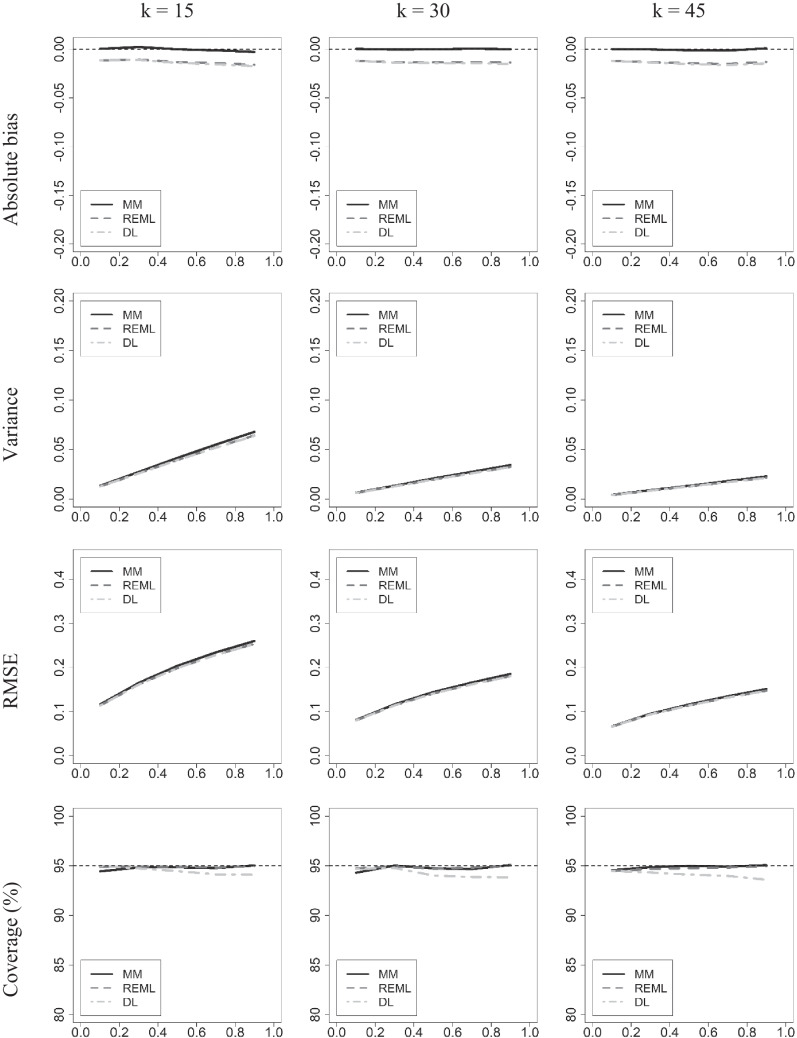
Performance of the estimator ${\widehat{\mu }}_{MM}$ as compared to both ${\widehat{\mu }}_{DL}$ and ${\widehat{\mu }}_{REML}$. The true value of *μ*_Δ_ is 0.50. In abscissa, the variance of true effects (*τ*^2^) is shown. The functions reflect the three estimators. In the columns are shown the three selected values of *k*

The results of the estimation of *μ*_*Δ*_ are shown in Fig. 3. We already know that our estimate is unbiased, but the results confirm this through a minimal empirical estimated bias, whereas REML and DL show a systematic but small bias. The efficiency and the RMSE are practically indistinguishable between the three methods. The 95% CI for *μ*_*Δ*_ has been obtained assuming a Student’s *t* distribution with (*k*-1) degrees of freedom and with the standard error estimated with (41). The coverages that appear in the last row of the figure show that the three methods achieve very good results. Estimates with MM and REML give coverages between 94 and 95% when *τ*^2^ is small (0.10) and practically 95% for higher values of the specific variance. Estimates with *DL* also performs well when the specific variance is small, and a little worse when is moderate or large, but still gives reasonably good coverages.

We have also wanted to empirically support the idea that the source of the bias in the estimation of *μ*_*δ*_ when using the REML and DL methods is the lack of independence between the *g* values and the weights calculated as the inverse of their estimated variances. To do this, we have calculated in each replica of each condition the Pearson’s correlation between the *g* values and the weights obtained as the inverse of the variances estimated by the MM, REML, and DL methods. A negative correlation between *g* and the weights would yield underestimations of *μ*_*δ*_. In the REM*L* and *DL* methods, the variances are obtained by adding the estimate of *τ*^2^ plus the estimate of the sampling variance with the unbiased formula of Hedges (1983; see Suero et al., 2023). After the 20,000 replicas, in all conditions of the simulation the average correlation with the MM weights are practically equal to 0 (the first figure different from 0 is in the third or fourth decimal position among the conditions simulated). On the contrary, the average correlations with the weights obtained with the REML and DL methods are always negative, as expected, and their values range from – 0.084 to – 0.172. These small but consistent negative correlations are what give rise to the systematic underestimation of *μ*_*δ*_ shown in the first row of Fig. 3 for the REML and DL methods.

Negative *g*-weight correlations have often been interpreted as indications of publication bias. While it is true that publication bias generates negative correlations, it is also true that their presence has low specificity as an indicator of this bias, given that they can occur for multiple reasons (Kühberger et al., 2014; Linden et al., 2024). In this simulation, we have shown that in the absence of publication bias small negative correlations occur simply by using an inadequate study variance. By the way, the size of the correlations estimated in our study are of similar size to those reported on real meta-analysis databases from various fields of psychology (Linden et al., 2024). At least as far as the standardized mean difference is concerned, negative correlations between *g* values and their estimated variances could be due to inadequate estimation of the variance of *g* at the study level. The same probably happens with other effect size indices whose variance depends on the value of the parameter itself.

Our conclusion from the simulation study is that, at least in the conditions assessed, the MM captures well the behavior of *g* and allows us to obtain unbiased estimators of *μ*_*Δ*_ and *τ*^2^. The advantage of ${\widehat{\mu }}_{\Delta (MM)}^{2}$ in terms of bias is not offset by advantages of the other two estimators in any of the other dimensions analyzed. The estimator ${\widehat{\mu }}_{\Delta (MM)}^{2}$ has practically optimal coverage, only with a very small margin of improvement in the condition with the smallest *k*.

The assessment of ${\widehat{\tau }}_{MM}^{2}$ is more complex. The most important characteristic of an estimator of this parameter should be the degree of bias. The estimator ${\widehat{\tau }}_{MM}^{2}$ clearly surpasses the other two in this sense, as it lacks bias, while the other two show significant degrees of bias. The degree of bias is somewhat higher with higher values of *k*, but above all, it increases when the value of the parameter itself increases, especially in the case of $\widehat\tau_{DL}^2$. It is true that the variance of both $\widehat\tau_{REML}^2$ and ${\widehat{\tau }}_{DL}^{2}$ are smaller than that of ${\widehat{\tau }}_{MM}^{2}$, but an advantage in efficiency does not compensate for a significant disadvantage in bias if the accompanying coverage of the confidence interval is too small. The coverage of the bootstrap estimations is not good with any method, but is better with ${\widehat{\tau }}_{MM}^{2}$. Clearly, we need an estimator of the variance of ${\widehat{\tau }}_{MM}^{2}$, and also to know its distribution. Only in this way can we build more reliable confidence intervals than those produced by the nonparametric bootstrap that we have used in the simulation.

In Appendix S5 (supplemental material) we include an R code segment for calculating our estimates. Appendix S6 (supplemental material) includes two examples with 20 and 10 studies, respectively. The code also calculates the estimates through the DL and REML methods with *metafor* (Viechtbauer, 2010) to notice the differences. Table 2 shows that in both examples, the estimates of the two parameters are slightly smaller with the REML and DL methods than with our MM estimators, as expected from the results of the previous simulation. The only slightly larger difference appears between ${\widehat{\tau }}_{MM}^{2}$ and the other two (${\widehat{\tau }}_{DL}^{2}$ and ${\widehat{\tau }}_{REML}^{2}$) in example 2.

**Table 2:** Estimates of the mean and variance of true effects with the three methods compared in two sets of studies (see Appendix S6)

	${\widehat{\mu }}_{\Delta }$	${\widehat{\tau }}^{2}$
Example 1 MM	0.3912	0.0870
REML	0.3819	0.0855
DL	0.3817	0.0781
Example 2 MM	0.6893	0.1508
REML	0.6467	0.0901
DL	0.6497	0.0965

## Discussion

The classic meta-analytic REM, formulated as a linear model, has guided technical developments since the work of Hedges (1981, 1983; Hedges & Olkin, 1985). Since the 1980s, most efforts have been devoted to the estimation issue. After all, the main goal of the applied meta-analyst is obtaining good estimates for the mean and variance of the true effects. Much less effort has been devoted to the assessment and refinement of the REM model itself. Beyond some isolated contributions, only recently have more adequate alternatives been proposed to address the weaknesses of the REM, despite the fact that some of the difficulties have been highlighted for decades, including the work of Hedges himself. This lack of effort is probably due to the assumption that the effects of the approximations and simplifications are small, or even negligible in practical terms.

A main difficulty for the REM has been the way in which it has been applied to the standardized mean difference (*g*), exposing a fundamental flaw. Essentially, the variance of the estimate from each study is obtained as ${\widehat{\tau }}^{2}$ plus the conditional sampling variance, given a specific value of the parameter, instead as the expected value of the conditional variance. Additionally, the formulas of the conditional variance generate an unnecessary, artificial covariation between the estimates and their variances. This means that, although the two random variables with which the classic model is represented (*u*_*i*_ and *e*_*i*_) are linearly independent, they are not stochastically independent, so the distribution of *g* is not the one indicated in most sources (Jackson & White, 2018; Lin & Aloe, 2021). The same problem can show up with other ES indices in which the conditional variance of the estimates of the individual studies involves the own estimated parameter of those studies.

These and other difficulties motivate a reformulation of the REM as a mixture model, in which the conditional variances of the estimates are not needed to estimate the variance of the marginal distribution of *g*, nor are they necessary to estimate the specific variance, *τ*^2^. Then, the formulas in this model are not affected by the main weakness of the classic REM. Our formulas for the mean, the variance and the skewness of the distribution of *g*, are very accurate. Furthermore, in contrast to the estimator most often used (DerSimonian Laird) and the most often recommended (REML; Viechtbauer, 2005), our estimator of *τ*^2^ is unbiased, although the efficiency and RMSE are comparable.

It can be argued that the practical differences between the MM and the classical REM model, especially estimating with REM*L*, are negligible. However, we have shown, for example, that the distribution of *g* is skewed (therefore, it is not a normal variable), so all the estimation methods of *τ*^2^ that assume normality are incorrect. However, the magnitude of the error is small, since the skewness is generally small. There are several methods for estimating the specific variance that assume normality in *g*. Despite not being precise, the performance of these estimators is quite good (see Langan et al., 2019; Veroniki et al., 2016), so there is little room for improvement in new estimators. Notably, the performance of the Malzahn et al. (2000) estimator, based on a formally identical mixture model as the one developed here, is comparable to that of the best estimators but without incorporating the unfounded assumption of normality (Blázquez-Rincón et al., 2023).

We believe that if one has a model that is formally correct it will always be better to use this model as a basis, rather than approximate, simplified models. Although the practical differences are small in the scenarios analyzed here, there may be other unexpected scenarios in which the differences are not so small. Furthermore, if other new developments are based on the approximate model, the deviations may be increasingly greater. For example, the present formulation could help to improve the methods to detect skewness in the funnel plot, a characteristic that is interpreted as evidence of publication bias. We have demonstrated that the true distribution of *g* is skewed, not normal. The skewness tests assess the null hypothesis of symmetry. So, if in the absence of publication bias the distribution does indeed have a slight positive skewness, it is to be expected that such tests will incur in an excess of type I errors. This is precisely what some simulation studies have shown (e.g., Carter et al., 2019; Zwetsloot et al., 2017). The skewness tests could be improved by taking into account the skewness of the distribution in the absence of publication bias and other contaminating factors.

There have been several attempts to solve the problems with *g* in the classic REM by changing the weighting scheme, for example, weighting by a function of the size of the sample(s), such as *N* or *ñ*. In this way, the weights do not correlate with the values of *g*, and also they are known, not estimated. However, there are two main reasons for preferring the inverse variance weighting. The first argument is that we do not give up searching for the minimum variance combination, in accordance with Cochran’s theorem. When weighting by a function of the sample sizes this objective is abandoned.

The second argument is that even if a solution for the weights based on a function of study size were achieved, we would still need the variances for other uses. For example, for inference about the mean effect size (*μ*_*δ*_): testing the null hypothesis *μ*_*δ*_ = 0, or building a confidence interval around the point estimate. Likewise, some procedures to test for symmetry in the funnel plot regresses the *g* values over their variances.

Using the (estimated) variances of the studies gives coherence to the meta-analytic methods and preserves the objective of searching for optimal weighting in the sense of minimum variance.

One of the problems that remains open is that of the distribution of the parametric value, *δ*_*i*_. The only assumption in this research is that the parametric value of the effect size is a continuous variable and that moments of order 1, 2, and 3 exist. Furthermore, the model has been developed for the case in which the distribution of the effect size estimator is also a continuous random variable.

Some alternatives have been proposed to provide an answer to this problem from a Bayesian approach, in which mixture models are also applied (e.g., Moreno et al., 2018), take the advantages of using Dirichlet processes (e.g., Karabatsos et al., 2015). Karabatsos et al. (2015) include moderators to solve the problem of assuming a particular distribution for parametric effect sizes. Given their Bayesian approach, they assume that the parameters involved in the model have associated a priori distributions (see Karabatsos et al., 2015, for a more detailed exposition). The model is defined in Eq. (7a) of Karabatsos et al., (2015, page 34). Comparing it with our Eq. (12), several important differences are observed. The main one is that in the case of Karabatsos et al. (2015), the variance of the marginal distribution is the sum of the variances of the distribution of the parametric effect sizes plus the variance (parametric or estimated) of the distribution of the estimator. However, we have shown that in the case that the latter depends on the parametric value of the effect size, this sum is not correct. Therefore, the Karabatsos et al. (2015) model should not be applied literally when the variance of the distribution of the effect size estimator depends on the parametric value. Its application to the standardized mean difference would suffer from the same fundamental flaw as the classic REM.

Moreno et al. (2018) approach the estimation process when random effects are assumed as a problem of grouping the primary studies included in a meta-analysis. In a meta-analysis made up of *k* estimates, two or more of these estimates belong to the same group (cluster) if they share the same parametric value of the effect size. Then, we would have to estimate, from the *k* values, the number of clusters and classify each of these values into a cluster. Although it is possible to theoretically imagine the scenario that motivates this model, in psychology it is not very credible, beyond the presence of categorical moderators, which are usually modeled as a fixed effect factor. In the absence of categorical moderators (or within the categories of such moderators) it is more reasonable to model psychological constructs with scenarios in which the probability of two studies sharing the same parametric value tends to zero. Rather, the values of *δ*_*i*_ represent variations around a general value, *μ*_*δ*_, which represents the overall magnitude of the effect.

Of course, the main limitation of this research is that we have not obtained good confidence intervals for the specific variance estimator. One of the next steps in the development of the MM should be to identify the shape of the distribution of ${\widehat{\tau }}_{MM}^{2}$. In this way, confidence intervals could be obtained with more accurate methods than the nonparametric bootstrap, and their coverage can be assessed more validly. This objective is at the top of our agenda for the future.

## Electronic supplementary material

Below is the link to the electronic supplementary material.

Supplementary file1 (DOCX 56 KB)

Supplementary file2 (DOCX 62 KB)

Supplementary file3 (DOCX 27 KB)

## Data Availability

Since Monte Carlo simulation is used as a methodology, re-executing any code segment will produce slightly different data.

## Appendix A

In this appendix, we specify some technical points related to *g* that we will refer to throughout the main text. The standardized mean difference (SMD) is defined as:

A1 $$\delta =({\mu }_{1}-{\mu }_{2})/\sigma$$

where μ_j_ is the mean of population *j*, whose distribution is normal, and *σ* is the common standard deviation of the two populations (two homoscedastic normal populations). With the data observed in two independent random samples of sizes *N*_1_ and *N*_2_, each coming from the respective population, the empirical *ES* index was defined by Cohen (1988) as:

A2 $$d=({\overline{X} }_{1}-{\overline{X} }_{2})/{S}_{pooled}$$

where ${\overline{X} }_{j}$ is the arithmetic mean of the sample of the population *j* and *S*_pooled_ is the standard deviation, combined through the variances of the two samples:

$${S}_{pooled}={\{[({N}_{1}-1)\cdot {S}_{1}^{2}+({N}_{2}-1)\cdot {S}_{2}^{2}]/({N}_{1}+{N}_{2}-2)\}}^{1/2}.$$

For *d* to be an unbiased estimator of *δ*, it must be corrected with Hedges' formula (1981), in which $c(m)=\Gamma (m/2)/\{\sqrt{m/2}\cdot \Gamma [(m-1)/2]\}$, Γ is the gamma function, and $m={N}_{1}+{N}_{2}-2$ reflects the degrees of freedom. The corrected *d* is usually designated as *g*:

A3 $$g=d\cdot c(m)$$

The expected value of *g* is *δ* (it is an unbiased estimator) and its variance is given by Hedges (1983, p. 390):

A4 $${\sigma }^{2}(g)=\frac{a}{\tilde{n} }[1+\tilde{n} {\delta }^{2}]-{\delta }^{2}$$

where $$\tilde{n} ={N}_{1}\cdot {N}_{2}/({N}_{1}+{N}_{2})$$ and $$a=m\cdot {[c(m)]}^{2}/(m-2)$$.

Then, (A4) is the variance of the distribution of *g*, given a fixed value of *δ* and a given pair of sample sizes (*N*_1_, *N*_2_). As *δ* is usually unknown, the variance of *g* must be estimated. Hedges (1983; p. 391) provides the following unbiased estimate of the variance of *g* for a given value of *δ*, although there are several alternative approximations (Suero et al., 2023):

A5 $${\widehat{\sigma }}_{1}^{2}(g)=\frac{1}{\tilde{n} }+(1-\frac{1}{a})\cdot {g}^{2}$$

The unbiased estimator, *g*, is a linear transformation that can be expressed as:

A6 $$g=c(m)\cdot \frac{1}{\sqrt{\tilde{n} }}\cdot t^{\prime}$$

where *t*′ is a random variable that follows a non-central Student's *t* distribution, with a non-centrality parameter equal to $\sqrt{\tilde{n} }\cdot \delta$, where *m* is the degrees of freedom. So, the moment of order *r* of *g* conditioned on *δ* is:

A7 $${\text{E}}({g}^{r}/\delta ;m,\widetilde{\text{n}})={\left(c(m)\cdot \frac{1}{\sqrt{\tilde{n} }}\right)}^{r}\cdot {\text{E}}(t{^{\prime}}^{r}/\delta )$$

And the moment of order *r*, ${\text{E}}(t{^{\prime}}^{r})$, is obtained by means of the expression (Johnson et al., 1994):

A8 $${\text{E}}({t}^{{\prime}r}/\delta )={\left(\frac{1}{2}\cdot m\right)}^{r/2}\cdot \frac{\Gamma \left(\frac{1}{2}\cdot (m-r)\right)}{\Gamma \left(\frac{1}{2}\cdot m\right)}\cdot \sum_{j=0}^{r/2}\left(\begin{array}{c}r\\ 2\cdot j\end{array}\right)\cdot \frac{\left(2\cdot j\right)!}{{2}^{j}\cdot j!}\cdot {\left(\sqrt{\widetilde{\text{n}}}\cdot \delta \right)}^{r-2\cdot j}$$

## Appendix B

Knowing that ${\sigma }_{G}^{2}+{\mu }_{\Delta }^{2}={\text{E}}({g}_{i}^{2})$, Eq. (38) can be expressed as:

B1 $${\tau }^{2}=\frac{k\cdot {\text{E}}({g}_{i}^{2})-\sum_{i=1}^{k}(\frac{{a}_{i}}{{\tilde{n} }_{i}})}{\sum_{i=1}^{k}{a}_{i}}-{\mu }_{\Delta }^{2}$$

On the other hand, given *k* primary studies, where study *i* contributes *g*_*i*_, the sample mean is a continuous random variable (CRV) with ${\text{E}}(\overline{g })={\mu }_{\Delta }$ and ${\sigma }_{\overline{g} }^{2}=\frac{{\sigma }_{G}^{2}}{k}$, where ${\sigma }_{G}^{2}$ is the variance of the marginal distribution of the standardized mean effect size estimator for variable sample sizes (see Eq. 32). Then,

B2 $${\text{E}}({\overline{g} }^{2})=\frac{\sum_{i=1}^{k}\frac{1}{k}\cdot (\frac{{a}_{i}}{{\tilde{n} }_{i}}+{a}_{i}({\tau }^{2}+{\mu }_{\Delta }^{2}))-{\mu }_{\Delta }^{2}}{k}+{\mu }_{\Delta }^{2}$$

Reordering:

B3 $${\text{E}}({\overline{g} }^{2})=\frac{\sum_{i=1}^{k}\frac{1}{k}\cdot (\frac{{a}_{i}}{{\tilde{n} }_{i}}+{a}_{i}({\tau }^{2}+{\mu }_{\Delta }^{2}))}{k}+\frac{{\mu }_{\Delta }^{2}\cdot (k-1)}{k}$$

And:

B4 $$\frac{k\cdot {\text{E}}({\overline{g} }^{2})-\sum_{i=1}^{k}\frac{{a}_{i}}{k\cdot {\tilde{n} }_{i}}}{\sum_{i=1}^{k}\frac{{a}_{i}}{k}}=({\tau }^{2}+{\mu }_{\Delta }^{2})+\frac{{\mu }_{\Delta }^{2}\cdot (k-1)}{\sum_{i=1}^{k}\frac{{a}_{i}}{k}}$$

Attending to (39) and remembering that ${\sigma }_{G}^{2}+{\mu }_{\Delta }^{2}={\text{E}}({g}_{i}^{2})$, then $({\tau }^{2}+{\mu }_{\Delta }^{2})=\frac{k\cdot {\text{E}}({g}_{i}^{2})-\sum_{i=1}^{k}(\frac{{a}_{i}}{{\tilde{n} }_{i}})}{\sum_{i=1}^{k}{a}_{i}}$.

Applying this equality to (B4):

B5 $${\mu }_{\Delta }^{2}=\frac{\sum_{i=1}^{k}\frac{{a}_{i}}{k}}{(k-1)}\cdot \left[\frac{k\cdot {\text{E}}({\overline{g} }^{2})-\sum_{i=1}^{k}\frac{{a}_{i}}{k\cdot {\tilde{n} }_{i}}}{\sum_{i=1}^{k}\frac{{a}_{i}}{k}}-\frac{k\cdot {\text{E}}({g}_{i}^{2})-\sum_{i=1}^{k}(\frac{{a}_{i}}{{\tilde{n} }_{i}})}{\sum_{i=1}^{k}{a}_{i}}\right]$$

Applying (B5) in (B1):

B6 $${\tau }^{2}=\frac{k\cdot {\text{E}}({g}_{i}^{2})-\sum_{i=1}^{k}\left(\frac{{a}_{i}}{{\widetilde{\text{n}}}_{i}}\right)}{\sum_{i=1}^{k}{a}_{i}}-\frac{\sum_{i=1}^{k}\frac{{a}_{i}}{k}}{(k-1)}\cdot [\frac{k\cdot {\text{E}}({\overline{g} }^{2})-\sum_{i=1}^{k}\frac{{a}_{i}}{k\cdot {\widetilde{\text{n}}}_{i}}}{\sum_{i=1}^{k}\frac{{a}_{i}}{k}}-\frac{k\cdot {\text{E}}({g}_{i}^{2})-\sum_{i=1}^{k}\left(\frac{{a}_{i}}{{\widetilde{\text{n}}}_{i}}\right)}{\sum_{i=1}^{k}{a}_{i}}]$$

Replacing $E={g}_{i}^{2}$ by $\sum_{i=1}^{k}{g}_{i}^{2}/k$, and $\text{E}\left({\overline{g} }^{2}\right)$ by ${\overline{g} }^{2}$, we reach the following formula:

B7 $${\widehat{\tau }}^{2}=\frac{\sum_{i=1}^{k}{g}_{i}^{2}-\sum_{i=1}^{k}\left(\frac{{a}_{i}}{{\widetilde{\text{n}}}_{i}}\right)}{\sum_{i=1}^{k}{a}_{i}}-\frac{\sum_{i=1}^{k}\frac{{a}_{i}}{k}}{\left(k-1\right)}\cdot [\frac{k\cdot {\overline{g} }^{2}-\sum_{i=1}^{k}\frac{{a}_{i}}{k\cdot {\widetilde{\text{n}}}_{i}}}{\sum_{i=1}^{k}\frac{{a}_{i}}{k}}-\frac{\sum_{i=1}^{k}{g}_{i}^{2}-\sum_{i=1}^{k}\left(\frac{{a}_{i}}{{\widetilde{\text{n}}}_{i}}\right)}{\sum_{i=1}^{k}{a}_{i}}]$$

where *k* is the number of primary studies and *g*_*i*_ is an unbiased estimate of the ES.

## Appendix C

In the formula for the variance of *g* at the study level (21b) appear both $({\tau }^{2}+{\mu }_{\Delta }^{2})$ and ${\mu }_{\Delta }^{2}$. Let's see how to estimate both expressions. With respect to $({\tau }^{2}+{\mu }_{\Delta }^{2})$, starting from formula (32) the following equality is reached:

C1 $$({\tau }^{2}+{\mu }_{\Delta }^{2})=\frac{k\cdot ({\sigma }_{G}^{2}+{\mu }_{\Delta }^{2})-\sum_{i=1}^{k}(\frac{{a}_{i}}{{\tilde{n} }_{i}})}{\sum_{i=1}^{k}{a}_{i}}$$

Since ${\text{E}\left({g}_{i}^{2}\right)=\sigma }_{G}^{2}+{\mu }_{\Delta }^{2}$ (remember that ${\mu }_{G}={\mu }_{\Delta }$), then (C1) is expressed as:

C2 $$({\tau }^{2}+{\mu }_{\Delta }^{2})=\frac{k\cdot {\text{E}}({g}_{i}^{2})-\sum_{i=1}^{k}(\frac{{a}_{i}}{{\tilde{n} }_{i}})}{\sum_{i=1}^{k}{a}_{i}}$$

From here, to estimate $({\tau }^{2}+{\mu }_{\Delta }^{2})$ it is replaced ${\text{E}}({g}_{i}^{2})$ by $\frac{\sum_{i=1}^{k}{g}_{i}^{2}}{k}$ and it is applied in (C2). Representing $({\tau }^{2}+{\mu }_{\Delta }^{2})$ by *P*, the estimator of that sum is:

C3 $$\widehat{P}=\frac{\sum_{i=1}^{k}{g}_{i}^{2}-\sum_{i=1}^{k}(\frac{{a}_{i}}{{\tilde{n} }_{i}})}{\sum_{i=1}^{k}{a}_{i}}$$

With respect to ${\mu }_{\Delta }^{2}$, plugging (C2) in (B4) we arrive at:

C4 $${\mu }_{\Delta }^{2}=\frac{\sum_{i=1}^{k}\frac{{a}_{i}}{k}}{(k-1)}\cdot [\frac{k\cdot {\text{E}}({\overline{g} }^{2})-\sum_{i=1}^{k}\frac{{a}_{i}}{k\cdot {\tilde{n} }_{i}}}{\sum_{i=1}^{k}\frac{{a}_{i}}{k}}-\frac{{\text{E}}({g}_{i}^{2})-\sum_{i=1}^{k}(\frac{{a}_{i}}{{\tilde{n} }_{i}})}{\sum_{i=1}^{k}{a}_{i}}]$$

Substituting $E({g}_{i}^{2})$ by $\sum_{i=1}^{k}{g}_{i}^{2}$, and $\text{E}\left({\overline{g} }^{2}\right)$ by ${\overline{g} }^{2}$, we arrive at the estimator:

C5 $${\widehat{\mu }}_{\Delta }^{2}=\frac{\sum_{i=1}^{k}\frac{{a}_{i}}{k}}{(k-1)}\cdot \left[\frac{k\cdot {\overline{g} }^{2}-\sum_{i=1}^{k}\frac{{a}_{i}}{k\cdot {\tilde{n} }_{i}}}{\sum_{i=1}^{k}\frac{{a}_{i}}{k}}-\frac{\sum_{i=1}^{k}{g}_{i}^{2}-\sum_{i=1}^{k}(\frac{{a}_{i}}{{\tilde{n} }_{i}})}{\sum_{i=1}^{k}{a}_{i}}\right]$$

## Code availability

Analysis and simulations code are available at https://doi.org/10.17605/OSF.IO/V2FDE

## Declarations

### Preregistration

This study was not pre-registered.

## References

[CR1] Bakbergenuly, I., Hoaglin, D. C., & Kulinskaya, E. (2020). Estimation in meta-analyses of mean difference and standardized mean difference. *Statistics in Medicine,**39*, 171–191. 10.1002/sim.8422 31709582 10.1002/sim.8422 PMC6916299

[CR2] Bakbergenuly, I., Hoaglin, D. C., & Kulinskaya, E. (2022). On the *Q* statistic with constant weights for standardized mean difference. *British Journal of Mathematical and Statistical Psychology,**75*(3), 444–465. 35094381 10.1111/bmsp.12263

[CR3] Beath, K. J. (2014). A finite mixture method for outlier detection and robustness in meta-analysis. *Research Synthesis Methods,**5*(4), 285–293. 26052953 10.1002/jrsm.1114

[CR4] Blázquez-Rincón, D., Sánchez-Meca, J., Botella, J., & Suero, M. (2023). Heterogeneity estimation in meta-analysis of standardized mean differences when the distribution of random effects departs from normal: A Monte Carlo simulation study. *BMC Medical Research Methodology,**23*(1), 19. 36650428 10.1186/s12874-022-01809-0 PMC9843903

[CR5] Böhning, D. (2000). *Computer-assisted Analysis of Mixtures**and Applications: Meta-analysis, Disease Mapping and Others* (Vol. 81). CRC Press.

[CR6] Böhning, D., Malzahn, U., Dietz, E., Schlattmann, P., Viwatwongkasem, C., & Biggeri, A. (2002). Some general points in estimating heterogeneity variance with the DerSimonian–Laird estimator. *Biostatistics,**3*(4), 445–457. 12933591 10.1093/biostatistics/3.4.445

[CR7] Borenstein, M., Hedges, L. V., Higgins, J. P. T., & Rothstein, H. R. (2010). A basic introduction to fixed-effects and random-effects models for meta-analysis. *Research Synthesis Methods,**1*, 97–111. 26061376 10.1002/jrsm.12

[CR8] Brannick, M. T., Potter, S., & Teng, Y. (2019). Quantifying uncertainty in the meta-analytic lower bound estimate. *Psychological Methods,**24*(6), 754. 31094545 10.1037/met0000217

[CR9] Buck, R. J., Fieberg, J., & Larkin, D. J. (2022). The use of weighted averages of Hedges’d in meta-analysis: Is it worth it? *Methods in Ecology and Evolution,**13*(5), 1093–1105.

[CR10] Carter, E. C., Schönbrodt, F. D., Gervais, W. M., & Hilgard, J. (2019). Correcting for bias in psychology: A comparison of meta-analytic methods. *Advances in Methods and Practices in Psychological Science,**2*(2), 115–144.

[CR11] Cochran, W. G. (1954). The combination of estimates from different experiments. *Biometrics,**10*, 101–129. 10.2307/3001666

[CR12] Cohen, J. (1988). *Statistical Power Analysis for the**Behavioural Scienc*es*, 2ª**ed*. New York: Academic Press.

[CR13] DerSimonian, R., & Laird, N. (1986). Meta-analysis in clinical trials. *Controlled Clinical Trials,**7*, 177–188. 10.1016/0197-2456(86)90046-2 3802833 10.1016/0197-2456(86)90046-2

[CR14] Doebler, P., & Holling, H. (2015). Meta-analysis of diagnostic accuracy and ROC curves with covariate-adjusted semiparametric mixtures. *Psychometrika,**80*(4), 1084–1104. 25361619 10.1007/s11336-014-9430-0

[CR15] Everitt, B. (2013). *Finite Mixture Distributions*. Springer Science & Business Media.

[CR16] Friedman, L. (2000). Estimators of random effects variance components in meta-analysis. *Journal of Educational and Behavioral Statistics,**25*(1), 1–12.

[CR17] Hamman, E. A., Pappalardo, P., Bence, J. R., Peacor, S. D., & Osenberg, C. W. (2018). *Bias in Meta-Analyses Using Hedges’d. Ecosphere,**9*(9), e02419.

[CR18] Hedges, L. V. (1981). Distribution theory for Glass’s estimator of effect size and related estimators. *Journal of Educational Statistics,**6*(2), 107–128.

[CR19] Hedges, L. V. (1982). Estimation of effect size from a series of independent experiments. *Psychological Bulletin,**92*(2), 490–499.

[CR20] Hedges, L. V. (1983). A random effects model for effect sizes. *Psychological Bulletin,**93*, 388–395. 10.1037/0033-2909.93.2.388

[CR21] Hedges, L. V. (2016). Comment on ‘Misunderstandings about Q and “Cochran’s Q test” in meta-analysis.’ *Statistics in Medicine,**35*(4), 496–497. 26776059 10.1002/sim.6763

[CR22] Hedges, L. V., & Olkin, I. (1985). *Statistical Methods for Meta-analysis*. Academic Press.

[CR23] Hoaglin, D. C. (2016). Misunderstandings about Q and ‘Cochran’s Q test’ in meta-analysis. *Statistics in Medicine,**35*(4), 485–495. 26303773 10.1002/sim.6632

[CR24] Holling, H., Böhning, W., & Böhning, D. (2012). Meta-analysis of diagnostic studies based upon SROC-curves: A mixed model approach using the Lehmann family. *Statistical Modelling,**12*(4), 347–375.

[CR25] Jackson, D., & White, I. R. (2018). When should meta-analysis avoid making hidden normality assumptions? *Biometrical Journal,**60*(6), 1040–1058. 30062789 10.1002/bimj.201800071 PMC6282623

[CR26] Johnson, N., Kotz, S, & Balakrishnan, N. (1994). *Continuous**Univariate**Distributions (2nd Ed., Vol. 2*). New York: Wiley.

[CR27] Karabatsos, G., Talbott, E., & Walker, S. G. (2015). A Bayesian nonparametric meta-analysis model. *Research Synthesis Methods,**6*(1), 28–44. 26035468 10.1002/jrsm.1117

[CR28] Kühberger, A., Fritz, A., & Scherndl, T. (2014). Publication bias in psychology: A diagnosis based on the correlation between effect size and sample size. *PLoS ONE,**9*(9), e105825. 25192357 10.1371/journal.pone.0105825 PMC4156299

[CR29] Kulinskaya, E., & Dollinger, M. B. (2016). Commentary on ‘Misunderstandings about Q and “Cochran’s Q test" in meta-analysis’. *Statistics in Medicine,**35*(4), 501–502. 26776061 10.1002/sim.6758

[CR30] Kulinskaya, E., Hoaglin, D. C., Bakbergenuly, I., & Newman, J. (2021). A Q statistic with constant weights for assessing heterogeneity in meta-analysis. *Research Synthesis Methods,**12*, 711–730. 10.1002/jrsm.1491 33969638 10.1002/jrsm.1491

[CR31] Langan, D., Higgins, J. P., Jackson, D., Bowden, J., Veroniki, A. A., Kontopantelis, E., ... & Simmonds, M. (2019). A comparison of heterogeneity variance estimators in simulated random‐effects meta‐analyses. *Research Synthesis Methods*, 10(1), 83–98. 10.1002/jrsm.1316 30067315

[CR32] Lin, L., & Aloe, A. M. (2021). Evaluation of various estimators for standardized mean difference in meta-analysis. *Statistics in Medicine,**40*, 403–426. 10.1002/sim.8781 33180373 10.1002/sim.8781 PMC7770064

[CR33] Linden, A. H., Pollet, T. V., & Hönekopp, J. (2024). Publication bias in psychology: A closer look at the correlation between sample size and effect size. *PLoS ONE,**19*(2), e0297075. 38359021 10.1371/journal.pone.0297075 PMC10868788

[CR34] Lindsay, B. G. (1995). Mixture models: Theory, geometry, and applications. *NSF-CBMS Regional Conference Series**in Probability and Statistics*, vol. 5. Institute of Mathematical Statistics, Hayward

[CR35] Malzahn, U., Böhning, D., & Holling, H. (2000). Nonparametric estimation of heterogeneity variance for the standardized difference used in meta-analysis. *Biometrika,**87*(3), 619–632.

[CR36] McLachlan, G. J., Lee, S. X., & Rathnayake, S. I. (2019). Finite mixture models. *Annual Review of Statistics and Its Application,**6*, 355–378.

[CR37] McLachlan, G., & Peel, D. (2000). *Finite Mixture Models*. Wiley.

[CR38] Moreno, E., Vázquez-Polo, F. J., & Negrín, M. A. (2018). Bayesian meta-analysis: The role of the between-sample heterogeneity. *Statistical Methods in Medical Research,**27*(12), 3643–3657. 28511616 10.1177/0962280217709837

[CR39] Petropoulou, M., & Mavridis, D. (2017). A comparison of 20 heterogeneity variance estimators in statistical synthesis of results from studies: A simulation study. *Statistics in Medicine,**36*(27), 4266–4280. 28815652 10.1002/sim.7431

[CR40] Shuster, J. J. (2016). Discussion of ‘Misunderstandings about Q and “Cochran’s Q test” in meta-analysis.’ *Statistics in Medicine,**4*(35), 498–500. 10.1002/sim.6762 26776060

[CR41] Suero, M., Botella, J., & Durán, J. I. (2023). Methods for estimating the sampling variance of the standardized mean difference. *Psychological Methods,**28*(4), 895–904. 34914477 10.1037/met0000446

[CR42] Thomas, H. (1989). A mixture model for distributions of correlation coefficients. *Psychometrika,**54*, 523–530.

[CR43] Titterington, D. M., Afm, S., Smith, A. F., & Makov, U. E. (1985). *Statistical Analysis of Finite Mixture Distributions* (Vol. 198). John Wiley & Sons Incorporated.

[CR44] Veroniki, A. A., Jackson, D., Viechtbauer, W., Bender, R., Bowden, J., Knapp, G., Kuss, O., Higgins, J. P. T., Langan, D., & Salanti, G. (2016). Methods to estimate the between study variance and its uncertainty in meta-analysis. *Research Synthesis Methods,**7*(1), 55–79. 26332144 10.1002/jrsm.1164 PMC4950030

[CR45] Viechtbauer, W. (2005). Bias and efficiency of meta-analytic variance estimators in the random-effects model. *Journal of Educational and Behavioral Statistics,**30*(3), 261–293.

[CR46] Viechtbauer, W. (2007). Confidence intervals for the amount of heterogeneity in meta-analysis. *Statistics in Medicine,**26*(1), 37–52. 16463355 10.1002/sim.2514

[CR47] Viechtbauer, W. (2010). Conducting meta-analyses in R with the *metafor* package. *Journal of Statistical Software,**36*(3), 1–48.

[CR48] Zwetsloot, P. P., Van Der Naald, M., Sena, E. S., Howells, D. W., IntHout, J., De Groot, J. A., ... & Wever, K. E. (2017). Standardized mean differences cause funnel plot distortion in publication bias assessments. *Elife*, 6, e24260. 10.7554/eLife.24260 PMC5621838 28884685

