# Supplementary material for: Reformulating the meta-analytical random effects model of the standardized mean difference as a mixture model
Source: Behav Res Methods. 2025 Jan 24;57(2):74. doi: 10.3758/s13428-024-02554-6 (PMC11761815; doi:10.3758/s13428-024-02554-6)
Supplement: Supplementary file 1 — Supplementary file1 (DOCX 56 KB) [file 13428_2024_2554_MOESM1_ESM.docx]

**Appendix S1**

The goal of the following demonstration is to show the consequences of the dependency between the parameter and its variance. To do this we show expressions for the variance of *v*, both when the variance of the *ES* does not depend on the value of *u* itself, and when it does depend.

If $v_{i}=u_{i}+e_{i}$, then the *pdf* of *v_i_* is:

$f_{V}(v)=\int_{-\infty}^{\infty} f_{U}(u;0,\tau)\cdot f_{E}(v-u;0,\sigma_{e})du$ (S1-1)

where E(*v*) = 0. The variance of *v* is:

$\sigma_{V}^{2}=\int_{-\infty}^{\infty} v^{2}\cdot f_{V}(v)dv=\int_{-\infty}^{\infty} v^{2}\int_{-\infty}^{\infty} f_{U}(u;0,\tau)\cdot f_{E}(v-u;0,\sigma_{e})dudv$ (S1-2)

Attending the right side of (S1-2) and the integral with respect to *v*, the following change of variable is made, $w=v-u$:

$\int_{-\infty}^{\infty} v^{2}\int_{-\infty}^{\infty} f_{U}(u;0,\tau)\cdot f_{E}(v-u;0,\sigma_{e})dudv=\int_{-\infty}^{\infty} (w+u)^{2}\int_{-\infty}^{\infty} f_{U}(u;0,\tau)\cdot f_{E}(w;0,\sigma_{e})dudw$(S1-3)

Rearranging terms and developing the binomial on the right side:

$\begin{matrix} \int_{-\infty}^{\infty} (w+u)^{2}\int_{-\infty}^{\infty} f_{U}(u;0,\tau)\cdot f_{E}(w;0,\sigma_{e})dudw=\int_{-\infty}^{\infty} \int_{-\infty}^{\infty} w^{2}\cdot f_{U}(u;0,\tau)\cdot f_{E}(w;0,\sigma_{e})dudw+ \\ \int_{-\infty}^{\infty} \int_{-\infty}^{\infty} u^{2}\cdot f_{U}(u;0,\tau)\cdot f_{E}(w;0,\sigma_{e})dudw+\int_{-\infty}^{\infty} \int_{-\infty}^{\infty} 2\cdot w\cdot u\cdot f_{U}(u;0,\tau)\cdot f_{E}(w;0,\sigma_{e})dudw \end{matrix}$(S1-4)

Since the expected values of *u_i_* and *w_i_* are both 0, the third addend is also 0. Actually, as the third addend is $\sigma_{WU}$, we conclude that the variables *w* and *u* are *linearly independent*.

With respect to the second addend, integrating with respect to *w* gives:

$\int_{-\infty}^{\infty} u^{2}f_{U}(u;0,\tau)\cdot du$ (S1-5)

That is $\tau^{2}$. Then the expression (S1-4) is equal to:

$\begin{matrix} \int_{-\infty}^{\infty} (w+u)^{2}\int_{-\infty}^{\infty} f_{U}(u;0,\tau)\cdot f_{E}(w;0,\sigma_{e})dudw= \\ =\tau^{2}+\int_{-\infty}^{\infty} \int_{-\infty}^{\infty} w^{2}\cdot f_{U}(u;0,\tau)\cdot f_{E}(w;0,\sigma_{e})dudw \end{matrix}$ (S1-6)

To solve the integrals in (S1-6) we must consider two cases. The first case occurs when $\sigma_{e}^{2}$ does not depend on the parametric value of the effect size. Then, the double integral is $\sigma_{e}^{2}$, and we obtain that the variance of *y* equals $\tau^{2}+\sigma_{e}^{2}$, which is the classical expression of the decomposition of the variance of *y* of formula (5).

The second case occurs when $\sigma_{e}^{2}$ depends on the parametric value. This happens in several *ES* indices, such as the *standardized mean difference*, *g* (Appendix A). In the case of *g*, for a given parametric value *δ* and samples sizes, the variance is equal to $\frac{a}{ñ}\left[1+ñ\delta^{2}\right]-\delta^{2}$ (Appendix A). Now, writing *δ* as in (2), $\mu_{\Delta}+u$, the variance of *g* is $\frac{a}{ñ}\left[1+ñ\cdot(\mu_{\Delta}+u)^{2}\right]-(\mu_{\Delta}+u)^{2}$. Therefore, there is a dependence between the variables; *u* and *w* are *linearly independent*, but they are *stochastically dependent*. Then, the variance of the random component of the model is:

$\sigma_{V}^{2}=\tau^{2}+\int_{-\infty}^{\infty} \int_{-\infty}^{\infty} w^{2}\cdot f_{U}(u;0,\tau)\cdot f_{E}(w/u;0,\sigma_{e}(u,\mu_{\Delta},m,ñ))dudw$ (S1-7)

It is clear in (S1-7) that $\sigma_{e}$ is a function of the crv *u*, the parameter $\mu_{\Delta}$, and the samples sizes involved in *m* and *ñ*. In order to make explicit the dependence of the variance of the aforementioned elements, $\sigma_{e}$ is expressed as $\sigma_{e}\left(u,\mu_{\Delta},m,ñ\right)$. Of course, the joint continuous distribution is $f_{U}(u;0,\tau)\cdot f_{E}(w/u;0,\sigma_{i}(u,\mu_{\Delta},m,ñ))$.

Integrating in (S1-7) with respect to the *crv* *w*, it is obtained the variance of *g* for a given value of *u*, of the parameter $\mu_{\Delta}$, and for the samples sizes involved in *m* and *ñ*. Then, the variance of *g* is:

$\begin{matrix} \sigma_{G}^{2}=\tau^{2}+\int_{-\infty}^{\infty} \int_{-\infty}^{\infty} w^{2}\cdot f_{U}(u;0,\tau)\cdot f_{E}(w/u;0,\sigma_{e}(u,\mu_{\Delta},m,ñ))dudw= \\ =\tau^{2}+\int_{-\infty}^{\infty} \left[\frac{a}{ñ}\left[1+ñ\cdot(\mu_{\Delta}+u)^{2}\right]-(\mu_{\Delta}+u)^{2}\right]\cdot f_{U}(u;0,\tau)\cdot du \end{matrix}$ (S1-8)

The second part to the right can be read as *the expected value of the conditional variances*. Then, the decomposition of the variance when the variance of *g* depends on the own parameter, *δ*, is not the classic expression as a sum of the variances of two independent random variables.
