# Supplementary material for: Reformulating the meta-analytical random effects model of the standardized mean difference as a mixture model
Source: Behav Res Methods. 2025 Jan 24;57(2):74. doi: 10.3758/s13428-024-02554-6 (PMC11761815; doi:10.3758/s13428-024-02554-6)
Supplement: Supplementary file 2 — Supplementary file2 (DOCX 62 KB) [file 13428_2024_2554_MOESM2_ESM.docx]

**Appendix S2**

It has been shown (see main text) that the *REM* can be defined as a *mixture model*:

$h_{G}(g;\mu_{\Delta},\tau,m,ñ)=\int_{-\infty}^{\infty} f_{G}(g/\delta;m,ñ)\cdot f_{\Delta}(\delta;\mu_{\Delta},\tau)d\delta$ (S2-1)

The centered moment of order *r* of *g* is defined as:

$\text{E}[(g-\mu_{G})^{r}]=\int_{-\infty}^{\infty} (g-\mu_{G})^{r}\int_{-\infty}^{\infty} f_{G}(g/\delta;m,ñ)\cdot f_{\Delta}(\delta;\mu_{\Delta},\tau)d\delta dg$ (S2-2)

Considering the Binomial Theorem[[1]](#footnote-1) (Hays, 1988, page 133) and rearranging:

$\text{E}[(g-\mu_{G})^{r}]=\int_{-\infty}^{\infty} \int_{-\infty}^{\infty} \sum_{k=0}^{r} (-1)^{k}\cdot\frac{r!}{k!\cdot(r-k)!}\cdot g^{r-k}\cdot\mu_{G}^{k}\cdot f_{G}(g/\delta;m,ñ)\cdot f_{\Delta}(\delta;\mu_{\Delta},\tau)dgd\delta$ (S2-3)

Integrating with respect to *g* in equation (S2-3) we obtain the moments, from order 0 to order *r*, of *g* conditioned on *δ*:

$\text{E}[(g-\mu_{G})^{r}]=\int_{-\infty}^{\infty} \sum_{k=0}^{r} (-1)^{k}\cdot\frac{r!}{k!\cdot(r-k)!}\cdot\left(c(m)\cdot\frac{1}{\sqrt{ñ}}\right)^{r-k}\text{·E}(t'^{ r-k}/\delta)\cdot\mu_{G}^{k}\cdot f_{\Delta}(\delta;\mu_{\Delta},\tau)d\delta$ (S2-4)

In the integral of (S2-4) only $\text{E}(t'^{ r-k}/\delta)$ and $f_{\Delta}(\delta;\mu_{\Delta},\tau)$depend on the variable of integration, *δ*, so (S2-4) can be expressed as:

$\text{E}[(g-\mu_{G})^{r}]=\sum_{k=0}^{r} (-1)^{k}\cdot\frac{r!}{k!\cdot(r-k)!}\cdot\left(c(m)\cdot\frac{1}{\sqrt{ñ}}\right)^{r-k}\cdot\mu_{\Delta}^{k}\cdot\int_{-\infty}^{\infty} \text{E}(t'^{ r-k}/\delta)\cdot f_{\Delta}(\delta;\mu_{\Delta},\tau)d\delta$ (S2-5)

To obtain the centered moment of order *r*, it is necessary to solve the integral:

$\int_{-\infty}^{\infty} \left(\frac{1}{2}\cdot m\right)^{(r-k)/2}\cdot\frac{\Gamma(\frac{1}{2}\cdot(m-(r-k)))}{\Gamma(\frac{1}{2}\cdot m)}\cdot\sum_{j=0}^{(r-k)/2} \left(\begin{matrix} r-k \\ 2\cdot j \end{matrix}\right)\cdot\frac{(2\cdot j)!}{2^{j}\cdot j!}\cdot\left(\sqrt{ñ}\cdot\delta\right)^{(r-k)-2\cdot j}f_{\Delta}(\delta;\mu_{\Delta},\tau)d\delta$(S2-6)

Taking into account the integration variable:

$\left(\frac{1}{2}\cdot m\right)^{(r-k)/2}\cdot\frac{\Gamma(\frac{1}{2}\cdot(m-(r-k)))}{\Gamma(\frac{1}{2}\cdot m)}\cdot\sum_{j=0}^{(r-k)/2} \left(\begin{matrix} r-k \\ 2\cdot j \end{matrix}\right)\cdot\frac{(2\cdot j)!}{2^{j}\cdot j!}\cdot\left(\sqrt{ñ}\right)^{(r-k)-2\cdot j}\cdot\int_{-\infty}^{\infty} \delta^{(r-k)-2\cdot j}\cdot f_{\Delta}(\delta;\mu_{\Delta},\tau)d\delta$(S2-7)

Now, the integral is expressing the moments of order (*r – k*) – *2·j* of the *pdf* of the parametric values $\delta$ (or $f_{\Delta}$): $\text{E}(\delta^{(r-k)-2\cdot j})$. So, the integral in (S2-5) is equal to:

$\int_{-\infty}^{\infty} \text{E}(t`^{r-k}/\delta)\cdot f_{\Delta}(\delta;\mu_{\Delta},\tau)d\delta=\left(\frac{1}{2}\cdot m\right)^{(r-k)/2}\cdot\frac{\Gamma(\frac{1}{2}\cdot(m-(r-k))}{\Gamma(\frac{1}{2}\cdot m)}\cdot\sum_{j=0}^{(r-k)/2} \left(\begin{matrix} r-k \\ 2\cdot j \end{matrix}\right)\cdot\frac{(2\cdot j)!}{2^{j}\cdot j!}\cdot\left(\sqrt{ñ}\right)^{(r-k)-2\cdot j}\cdot\text{E}(\delta^{(r-k)-2\cdot j})$(S2-8)

Substituting the result of this integral in (S2-5) we obtain:

$$\text{E}[(g-\mu_{G})^{r}]=\sum_{k=0}^{r} (-1)^{k}\cdot\frac{r!}{k!\cdot(r-k)!}\cdot\left(c(m)\cdot\frac{1}{\sqrt{ñ}}\right)^{r-k}\cdot\mu_{G}^{k}\cdot\left(\frac{1}{2}\cdot m\right)^{r/2}\cdot\frac{\Gamma(\frac{1}{2}\cdot(m-(r-k)))}{\Gamma(\frac{1}{2}\cdot m)}\cdot\sum_{j=0}^{(r-k)/2} \left(\begin{matrix} r-k \\ 2\cdot j \end{matrix}\right)\cdot\frac{(2\cdot j)!}{2^{j}\cdot j!}\cdot\left(\sqrt{ñ}\right)^{(r-k)-2\cdot j}\cdot\text{E}(\delta^{(r-k)-2\cdot j})$$

(S2-9)

This expression allows us to obtain the expected value centered and also that of order *r* of the *pdf* $h_{G}$. Implied in the expression is the expected value of *g*, or $\mu_{G}$. Specifically:

$\mu_{G}=\int_{-\infty}^{\infty} g\cdot\int_{-\infty}^{\infty} f_{G}(g/\delta;m,ñ)\cdot f_{\Delta}(\delta;\mu_{\Delta},\tau)d\delta dg$ (S2-10)

Considering the integral with respect to *g*, it can be seen that it is the definition of the expected value of $f_{G}(g/\delta;m,ñ)$, which is *δ* (remember that *g* is an unbiased estimator of *δ*), so that:

$\mu_{G}=\int_{-\infty}^{\infty} \delta\cdot f_{\Delta}(\delta;\mu_{\Delta},\tau)d\delta$ (S2-11)

This integral is the expected value of the *pdf* $f_{\Delta}$, so:

$\mu_{G}=\mu_{\Delta}$ (S2-12)

Substituting the result of this integral in (S2-9):

$$\text{E}[(g-\mu_{\Delta})^{r}]=\sum_{k=0}^{r} (-1)^{k}\cdot\frac{r!}{k!\cdot(r-k)!}\cdot\left(c(m)\cdot\frac{1}{\sqrt{ñ}}\right)^{r-k}\cdot\mu_{\Delta}^{k}\cdot\left(\frac{1}{2}\cdot m\right)^{r/2}\cdot\frac{\Gamma(\frac{1}{2}\cdot(m-(r-k)))}{\Gamma(\frac{1}{2}\cdot m)}\cdot\sum_{j=0}^{(r-k)/2} \left(\begin{matrix} r-k \\ 2\cdot j \end{matrix}\right)\cdot\frac{(2\cdot j)!}{2^{j}\cdot j!}\cdot\left(\sqrt{ñ}\right)^{(r-k)-2\cdot j}\cdot\text{E}(\delta^{(r-k)-2\cdot j})$$

(S2-13)

1. The binomial theorem (Hays, 1988): $(x-y)^{n}=\sum_{k=0}^{n} (-1)^{k}\cdot\frac{n!}{k!\cdot(n-k)!}\cdot x^{n-k}\cdot y^{k}$ [↑](#footnote-ref-1)
