# Supplementary material for: Reformulating the meta-analytical random effects model of the standardized mean difference as a mixture model
Source: Behav Res Methods. 2025 Jan 24;57(2):74. doi: 10.3758/s13428-024-02554-6 (PMC11761815; doi:10.3758/s13428-024-02554-6)
Supplement: Supplementary file 3 — Supplementary file3 (DOCX 27 KB) [file 13428_2024_2554_MOESM3_ESM.docx]

**APPENDIX S3**

Segment of R code of the simulation in section 6 (table 1)

sim_data_d <- function(mu_delta, tau, n1, n2, delta_theor_distrib = "norm"){

if(length(n1) != length(n2)){

stop("n1 and n2 must have the same length (k)")

}

data <- array(NA, dim = c(length(n1), 18))

colnames(data) <- c("study", "mu_delta", "tau", "sample_s", "n1", "n2", "m", "nn", "cm", "a", "delta", "d", "g", "var_g", "m1i", "m2i", "sd1i", "sd2i")

for(i in 1:length(n1)){

x_control <- rnorm(n = n1[i], mean = 0, sd = 1)

delta <- get(paste("r", delta_theor_distrib, sep = ""))(1, mu_delta, tau)

x_exp <- rnorm(n = n2[i], mean = delta, sd = 1)

descriptives <- c(mean(x_control), var(x_control), mean(x_exp), var(x_exp))

names(descriptives) <- c("mControl", "varControl", "mExp", "varExp")

m1i <- descriptives["mControl"]

m2i <- descriptives["mExp"]

sd1i <- sqrt(descriptives["varControl"])

sd2i <- sqrt(descriptives["varExp"])

m <- n1[i] + n2[i] - 2

nn <- (n1[i] * n2[i]) / (n1[i] + n2[i])

cm <- gamma(m / 2) / ( (sqrt(m / 2)) * gamma((m - 1) / 2) )

a <- (m * (cm^2)) / (m - 2)

vart <- ( ((n1[i] - 1) * descriptives["varControl"]) + ((n2[i] - 1) * descriptives["varExp"]) ) / m

d <- ( (descriptives["mExp"] - descriptives["mControl"]) / sqrt(vart) )

g <- d * cm

var_g <- (1 / nn) + ((1 - (1/a)) * (g^2)) # Formula 9 Hedges (1983)

data[i,] <- c(i, mu_delta, tau, n1[i]+n2[i], n1[i], n2[i], m, nn, cm, a, delta, d, g, var_g, m1i, m2i, sd1i, sd2i)

}

return(data)

}

mu_delta <- 0.5

tau2 <- 0.1

k <- 10^7

sample_s_fixed <- rep(30 / 2, 2)

sample_s_variable <- cbind(

c(5, 5, 10, 10, 15, 10, 15, 20),

c(5, 15, 10, 20, 15, 30, 25, 20)

)

# Fixed n1 and n2

a_fixed <- c()

for(i in 1:k){

a_fixed <- rbind(a_fixed, sim_data_d(mu_delta = mu_delta, tau = sqrt(tau2), n1 = sample_s_fixed[1], n2 = sample_s_fixed[2]))

}

sigma2_g_fixed <- ( ( ((a_fixed[1,"cm"] ^ 2) * a_fixed[1,"m"]) / ((a_fixed[1,"m"] - 2) * a_fixed[1,"nn"]) ) * (1 + (a_fixed[1,"nn"] * (tau2 + (mu_delta^2)))) ) - (mu_delta^2) # Theoretical variance of g_i (mixture model, fixed N)

res_fixed <- c(

mean(a_fixed[,"g"]), var(a_fixed[,"g"]), mean( ((a_fixed[,"g"] - mean(a_fixed[,"g"])) / sd(a_fixed[,"g"])) ^3 ), # empirical mean, variance and skewness of g

mu_delta, #Theoretical mean

mean(sigma2_g_fixed), #Theoretical variance

mean(

( (a_fixed[,"cm"] ^ 3) / a_fixed[,"nn"] ) *

( ( (a_fixed[,"m"] ^ (3/2)) * gamma((a_fixed[,"m"] - 3) / 2) ) / (2 * sqrt(2) * gamma(a_fixed[,"m"] / 2)) ) *

(3 * mu_delta + ( mu_delta * ((mu_delta^2) + (3 * tau2)) ) * a_fixed[,"nn"]) -

(3 * mu_delta) * sigma2_g_fixed - (mu_delta ^ 3)

) / (sqrt(mean(sigma2_g_fixed)) ^ 3) # Theoretical skewness

)

names(res_fixed) <- c("mu_delta_emp", "var_emp", "skew_emp", "mu_delta_mix", "var_g_mix", "skew_g_mix")

# Variable values of n1 and n2

a_variable <- c()

for(i in 1:k){

a_variable <- rbind(a_variable, sim_data_d(mu_delta = mu_delta, tau = sqrt(tau2), n1 = sample_s_variable[,1], n2 = sample_s_variable[,2]))

}

sigma2_g_variable <- ( ((a_variable[,"cm"] ^ 2) * a_variable[,"m"]) / ((a_variable[,"m"] - 2) * a_variable[,"nn"]) ) * ( 1 + a_variable[,"nn"] * (tau2 + (mu_delta^2))) - (mu_delta^2) # Variance mixture model (variable N)

res_variable <- c(

mean(a_variable[,"g"]), var(a_variable[,"g"]), mean( ((a_variable[,"g"] - mean(a_variable[,"g"])) / sd(a_variable[,"g"])) ^3 ), # Empirical mean, variance and skewness of g

mu_delta, #Theoretical mean

mean(sigma2_g_variable), #Theoretical variance

mean(

( (a_variable[,"cm"] ^ 3) / a_variable[,"nn"] ) *

( ( (a_variable[,"m"] ^ (3/2)) * gamma((a_variable[,"m"] - 3) / 2) ) / (2 * sqrt(2) * gamma(a_variable[,"m"] / 2)) ) *

(3 * mu_delta + ( mu_delta * ((mu_delta^2) + (3 * tau2)) ) * a_variable[,"nn"]) -

(3 * mu_delta) * sigma2_g_variable - (mu_delta ^ 3)

) / (sqrt(mean(sigma2_g_variable)) ^ 3) # Theoretical skewness

)

names(res_variable) <- c("mu_delta_emp", "var_emp", "skew_emp", "mu_delta_mix", "var_g_mix", "skew_g_mix")

# Mean, variance and skewness of g (empirical, theoretical and differences)

res_fixed

res_fixed["mu_delta_mix"] - res_fixed["mu_delta_emp"]

res_fixed["var_g_mix"] - res_fixed["var_emp"]

res_fixed["skew_g_mix"] - res_fixed["skew_emp"]

res_variable

res_variable["mu_delta_mix"] - res_variable["mu_delta_emp"]

res_variable["var_g_mix"] - res_variable["var_emp"]

res_variable["skew_g_mix"] - res_variable["skew_emp"]

**APPENDIX S4**

Segment of R code that performs the simulation of section 7

rm(list=ls(all=T))

library("parallel")

library("foreach")

library("doParallel")

## Function for estimating mu_delta and tau2 with Mixture Model, Restricted Maximum Likelihood (REML) and Dersimonian-Laird (DL) methods

estim <- function(ES_i, n1, n2, ES_index = "g", boot_samples = 10000){

library("metafor")

m <- n1 + n2 - 2

nn <- (n1 * n2) / (n1 + n2)

cm <- gamma(m / 2) / ( (sqrt(m / 2)) * gamma((m - 1) / 2) )

a <- (m * (cm^2)) / (m - 2)

if(ES_index == "g"){

g <- ES_i

}

if(ES_index == "d"){

g <- ES_i * cm

}

tau2_estim_MM <- ( (sum(g^2) - sum(a / nn)) / sum(a) ) -

( sum(a / length(g)) / (length(g) - 1) ) *

(

( ( length(g) * (mean(g)^2) - sum(a / (length(g) * nn)) ) / sum(a / length(g)) ) -

(sum(g^2) - sum(a / nn)) / sum(a)

)

if(tau2_estim_MM > 0){

sigma_g_estim <- (a / nn) + a * ((sum(g^2) - sum(a/nn)) / sum(a)) -

(

(sum(a/length(g)) / (length(g)-1)) *

( ( ( length(g) * mean(g)^2 - sum(a/(length(g)*nn)) ) / sum(a/length(g)) ) -

((sum(g^2) - sum(a/nn)) / sum(a)) )

)

} else {

tau2_estim_MM <- 0

sigma_g_estim <- (1 / nn) + ((1 - (1/a)) * ((sum(nn*g)/sum(nn))^2)) # Hedges (1983) (with ñ weighted delta estimate)

}

var_g <- (1 / nn) + ((1 - (1/a)) * (g^2)) # Hedges (1983)

mu_delta_estim_MM <- sum((1 / sigma_g_estim) * g) / sum(1 / sigma_g_estim)

amp_t <- sqrt((1 / sum(1 / sigma_g_estim))) * qt(0.975, df = length(g)-1)

mu_delta_lb <- mu_delta_estim_MM - amp_t

mu_delta_ub <- mu_delta_estim_MM + amp_t

REML <- rma.uni(yi = g, vi = var_g, method = "REML", test = "t") #Restricted Maximum Likelihood

DL <- rma.uni(yi = g, vi = var_g, method = "DL", test = "t") #Dersimonian-Laird

# Tau2 CI by bootstrap sampling

tau2_boot <- matrix(NA, boot_samples, 3)

colnames(tau2_boot) <- c("tau2_MM_i", "tau2_REML_i", "tau2_DL_i")

for(i in 1:boot_samples){ # bootstrap loop

samp_i <- sample(1:length(g), replace = T)

data_samp_i <- cbind(g, n1, n2, nn, a, var_g)[samp_i,]

colnames(data_samp_i) <- c("g", "n1", "n2", "nn", "a", "var_g")

tau2_MM_i <- ( (sum(data_samp_i[,"g"]^2) - sum(data_samp_i[,"a"] / data_samp_i[,"nn"])) / sum(data_samp_i[,"a"]) ) -

( sum(data_samp_i[,"a"] / nrow(data_samp_i)) / (nrow(data_samp_i) - 1) ) *

(

( ( nrow(data_samp_i) * (mean(data_samp_i[,"g"])^2) - sum(data_samp_i[,"a"] / (nrow(data_samp_i) * data_samp_i[,"nn"])) ) / sum(data_samp_i[,"a"] / nrow(data_samp_i)) ) -

(sum(data_samp_i[,"g"]^2) - sum(data_samp_i[,"a"] / data_samp_i[,"nn"])) / sum(data_samp_i[,"a"])

)

tau2_MM_i <- ifelse(tau2_MM_i > 0, tau2_MM_i, 0)

tau2_REML_i <- tryCatch(

{rma.uni(yi = data_samp_i[,"g"], vi = data_samp_i[,"var_g"], method = "REML", test = "t", control = list(maxiter = 200))$tau2}, #Restricted Maximum Likelihood

error = function(x){return(NA)}

)

tau2_DL_i <- tryCatch(

{rma.uni(yi = data_samp_i[,"g"], vi = data_samp_i[,"var_g"], method = "DL", test = "t", control = list(maxiter = 200))$tau2}, #Dersimonian-Laird

error = function(x){return(NA)}

)

tau2_boot[i,] <- c(tau2_MM_i, tau2_REML_i, tau2_DL_i)

}

tau2_CI_MM <- quantile(tau2_boot[,"tau2_MM_i"], probs = c(0.025, 0.975), na.rm = T)

tau2_CI_REML <- quantile(tau2_boot[,"tau2_REML_i"], probs = c(0.025, 0.975), na.rm = T)

tau2_CI_DL <- quantile(tau2_boot[,"tau2_DL_i"], probs = c(0.025, 0.975), na.rm = T)

res <- c(mu_delta_estim_MM, mu_delta_lb, mu_delta_ub, tau2_estim_MM, tau2_CI_MM,

REML$beta, REML$ci.lb, REML$ci.ub, REML$tau2, tau2_CI_REML,

DL$beta, DL$ci.lb, DL$ci.ub, DL$tau2, tau2_CI_DL)

names(res) <- c("mu_delta_estim_MM", "mu_delta_estim_MM_lb", "mu_delta_estim_MM_ub", "tau2_estim_MM", "tau2_estim_MM_lb", "tau2_estim_MM_ub",

"REML_mu_delta", "REML_mu_delta_lb", "REML_mu_delta_ub", "REML_tau2", "REML_tau2_lb", "REML_tau2_ub",

"DL_mu_delta", "DL_mu_delta_lb", "DL_mu_delta_ub", "DL_tau2", "DL_tau2_lb", "DL_tau2_ub")

return(res)

}

## Function for data generation

sim_data_d <- function(mu_delta, tau, n1, n2, delta_theor_distrib = "norm"){

if(length(n1) != length(n2)){

stop("n1 and n2 must have the same length (k)")

}

data <- array(NA, dim = c(length(n1), 18))

colnames(data) <- c("study", "mu_delta", "tau", "sample_s", "n1", "n2", "m", "nn", "cm", "a", "delta", "d", "g", "var_g", "m1i", "m2i", "sd1i", "sd2i")

for(i in 1:length(n1)){

x_control <- rnorm(n = n1[i], mean = 0, sd = 1)

delta <- get(paste("r", delta_theor_distrib, sep = ""))(1, mu_delta, tau)

x_exp <- rnorm(n = n2[i], mean = delta, sd = 1)

descriptives <- c(mean(x_control), var(x_control), mean(x_exp), var(x_exp))

names(descriptives) <- c("mControl", "varControl", "mExp", "varExp")

m1i <- descriptives["mControl"]

m2i <- descriptives["mExp"]

sd1i <- sqrt(descriptives["varControl"])

sd2i <- sqrt(descriptives["varExp"])

m <- n1[i] + n2[i] - 2

nn <- (n1[i] * n2[i]) / (n1[i] + n2[i])

cm <- gamma(m / 2) / ( (sqrt(m / 2)) * gamma((m - 1) / 2) )

a <- (m * (cm^2)) / (m - 2)

vart <- ( ((n1[i] - 1) * descriptives["varControl"]) + ((n2[i] - 1) * descriptives["varExp"]) ) / m

d <- ( (descriptives["mExp"] - descriptives["mControl"]) / sqrt(vart) )

g <- d * cm

var_g <- (1 / nn) + ((1 - (1/a)) * (g^2)) # Formula 9 Hedges (1983)

data[i,] <- c(i, mu_delta, tau, n1[i]+n2[i], n1[i], n2[i], m, nn, cm, a, delta, d, g, var_g, m1i, m2i, sd1i, sd2i)

}

return(data)

}

## Monte-Carlo simulation study

# Simulation factors

mu_delta <- seq(0.5, 0.5, 0)

tau2 <- seq(0.1, 0.9, 0.2)

k <- seq(15, 45, 15)

N <- cbind(

c(10, 5, 15, 10, 20, 10, 15, 25, 20, 40, 30, 50, 60),

c(10, 15, 15, 20, 20, 30, 25, 25, 30, 40, 50, 50, 40)

)

conditions <- expand.grid(mu_delta = mu_delta, tau2 = tau2, k = k)

replications <- 20000

#Parallel computation parameters

propcores <- 0.75

cores <- round(detectCores() * propcores)

registerDoParallel(cores)

#Data generation and estimators computation

res <- c()

des <- c()

primary <- c()

for(c in 1:nrow(conditions)){

res <- foreach(i = 1:replications, .combine = "rbind") %dopar% {

n_i <- N[sample(1:nrow(N), conditions[c, "k"], replace = TRUE), ]

sim <- sim_data_d(mu_delta = conditions[c, "mu_delta"], tau = sqrt(conditions[c, "tau2"]), n1 = n_i[, 1], n2 = n_i[, 2]) # Data generation

estim(ES_i = sim[,"g"], n1 = sim[,"n1"], n2 = sim[,"n2"]) # Computation of estimators from data

}

des <- rbind( des, c(conditions[c, "mu_delta"], conditions[c, "tau2"], conditions[c, "k"],

mean(res[,"mu_delta_estim_MM"] - conditions[c, "mu_delta"]), sum(ifelse(res[,"mu_delta_estim_MM_lb"] < conditions[c, "mu_delta"] & res[,"mu_delta_estim_MM_ub"] > conditions[c, "mu_delta"], 1, 0)) / nrow(res),

mean(res[,"tau2_estim_MM"] - conditions[c, "tau2"]), sum(ifelse(res[,"tau2_estim_MM_lb"] < conditions[c, "tau2"] & res[,"tau2_estim_MM_ub"] > conditions[c, "tau2"], 1, 0)) / nrow(res),

var(res[,"mu_delta_estim_MM"]), var(res[,"tau2_estim_MM"]),

sqrt(mean((res[,"mu_delta_estim_MM"] - conditions[c, "mu_delta"])^2)), sqrt(mean((res[,"tau2_estim_MM"] - conditions[c, "tau2"])^2)),

mean(res[,"REML_mu_delta"] - conditions[c, "mu_delta"]), sum(ifelse(res[,"REML_mu_delta_lb"] < conditions[c, "mu_delta"] & res[,"REML_mu_delta_ub"] > conditions[c, "mu_delta"], 1, 0)) / nrow(res),

mean(res[,"REML_tau2"] - conditions[c, "tau2"]), sum(ifelse(res[,"REML_tau2_lb"] < conditions[c, "tau2"] & res[,"REML_tau2_ub"] > conditions[c, "tau2"], 1, 0)) / nrow(res),

var(res[,"REML_mu_delta"]), var(res[,"REML_tau2"]),

sqrt(mean((res[,"REML_mu_delta"] - conditions[c, "mu_delta"])^2)), sqrt(mean((res[,"REML_tau2"] - conditions[c, "tau2"])^2)),

mean(res[,"DL_mu_delta"] - conditions[c, "mu_delta"]), sum(ifelse(res[,"DL_mu_delta_lb"] < conditions[c, "mu_delta"] & res[,"DL_mu_delta_ub"] > conditions[c, "mu_delta"], 1, 0)) / nrow(res),

mean(res[,"DL_tau2"] - conditions[c, "tau2"]), sum(ifelse(res[,"DL_tau2_lb"] < conditions[c, "tau2"] & res[,"DL_tau2_ub"] > conditions[c, "tau2"], 1, 0)) / nrow(res),

var(res[,"DL_mu_delta"]), var(res[,"DL_tau2"]),

sqrt(mean((res[,"DL_mu_delta"] - conditions[c, "mu_delta"])^2)), sqrt(mean((res[,"DL_tau2"] - conditions[c, "tau2"])^2))

))

print(paste("mu_delta: ", conditions[c, "mu_delta"], "tau2: ", conditions[c, "tau2"], "| k: ", conditions[c, "k"]))

res <- c()

}

colnames(des) <- c("mu_delta", "tau2", "k",

"bias_mu_delta_MM", "coverage_mu_delta_MM",

"bias_tau2_MM", "coverage_tau2_MM",

"var_mu_delta_MM", "var_tau2_MM",

"RMSE_mu_delta_MM", "RMSE_tau2_MM",

"bias_mu_delta_REML", "coverage_mu_delta_REML",

"bias_tau2_REML", "coverage_tau2_REML",

"var_mu_delta_REML", "var_tau2_REML",

"RMSE_mu_delta_REML", "RMSE_tau2_REML",

"bias_mu_delta_DL", "coverage_mu_delta_DL",

"bias_tau2_DL", "coverage_tau2_DL",

"var_mu_delta_DL", "var_tau2_DL",

"RMSE_mu_delta_DL", "RMSE_tau2_DL"

)

des

**APPENDIX S5**

Segment of R code that yields the estimates of the mean (μΔ) and the variance (τ2) of the true effects under the MM

#"estim" function works providing g, n1 and n2 values for each primary study

# Values of d can be provided instead of the bias-corrected g index adding the optional argument "ES_index = "d"

estim <- function(ES_i, n1, n2, ES_index = "g", boot_samples = 10000){

m <- n1 + n2 - 2

nn <- (n1 * n2) / (n1 + n2)

cm <- gamma(m / 2) / ( (sqrt(m / 2)) * gamma((m - 1) / 2) )

a <- (m * (cm^2)) / (m - 2)

if(ES_index == "g"){

g <- ES_i

}

if(ES_index == "d"){

g <- ES_i * cm

}

tau2_estim_MM <- ( (sum(g^2) - sum(a / nn)) / sum(a) ) -

( sum(a / length(g)) / (length(g) - 1) ) *

(

( ( length(g) * (mean(g)^2) - sum(a / (length(g) * nn)) ) / sum(a / length(g)) ) -

(sum(g^2) - sum(a / nn)) / sum(a)

)

if(tau2_estim_MM > 0){

sigma_g_estim <- (a / nn) + a * ((sum(g^2) - sum(a/nn)) / sum(a)) -

(

(sum(a/length(g)) / (length(g)-1)) *

( ( ( length(g) * mean(g)^2 - sum(a/(length(g)*nn)) ) / sum(a/length(g)) ) -

((sum(g^2) - sum(a/nn)) / sum(a)) )

)

} else {

tau2_estim_MM <- 0

sigma_g_estim <- (1 / nn) + ((1 - (1/a)) * ((sum(nn*g)/sum(nn))^2)) # Hedges (1983) (with ñ weighted delta estimate)

}

mu_delta_estim_MM <- sum((1 / sigma_g_estim) * g) / sum(1 / sigma_g_estim)

amp_t <- sqrt((1 / sum(1 / sigma_g_estim))) * qt(0.975, df = length(g)-1)

mu_delta_lb <- mu_delta_estim_MM - amp_t

mu_delta_ub <- mu_delta_estim_MM + amp_t

# Tau2 CI by bootstrap sampling

tau2_boot <- c()

for(i in 1:boot_samples){ # bootstrap loop

samp_i <- sample(1:length(g), replace = T)

data_samp_i <- cbind(g, n1, n2, nn, a)[samp_i,]

colnames(data_samp_i) <- c("g", "n1", "n2", "nn", "a")

tau2_MM_i <- ( (sum(data_samp_i[,"g"]^2) - sum(data_samp_i[,"a"] / data_samp_i[,"nn"])) / sum(data_samp_i[,"a"]) ) -

( sum(data_samp_i[,"a"] / nrow(data_samp_i)) / (nrow(data_samp_i) - 1) ) *

(

( ( nrow(data_samp_i) * (mean(data_samp_i[,"g"])^2) - sum(data_samp_i[,"a"] / (nrow(data_samp_i) * data_samp_i[,"nn"])) ) / sum(data_samp_i[,"a"] / nrow(data_samp_i)) ) -

(sum(data_samp_i[,"g"]^2) - sum(data_samp_i[,"a"] / data_samp_i[,"nn"])) / sum(data_samp_i[,"a"])

)

tau2_MM_i <- ifelse(tau2_MM_i > 0, tau2_MM_i, 0)

tau2_boot <- c(tau2_boot, tau2_MM_i)

}

tau2_CI_MM <- quantile(tau2_boot, probs = c(0.025, 0.975), na.rm = T)

res <- c(mu_delta_estim_MM, mu_delta_lb, mu_delta_ub, tau2_estim_MM, tau2_CI_MM)

names(res) <- c("mu_delta_estim_MM", "mu_delta_estim_MM_lb", "mu_delta_estim_MM_ub", "tau2_estim_MM", "tau2_estim_MM_lb", "tau2_estim_MM_ub")

return(res)

}

**APPENDIX S6**

Segment of R code that provides the estimates of μΔ and τ2 under the MM, and also under the classical REM estimating the specific variance with the REML and DL methods with metafor. The estimating function is “MM estimators function.R” (appendix S5). It is prepared to process a file named "Data.xlxs"

library("readxl")

estim <- function(ES_i, n1, n2, ES_index = "g", boot_samples = 10000){

library("metafor")

m <- n1 + n2 - 2

nn <- (n1 * n2) / (n1 + n2)

cm <- gamma(m / 2) / ( (sqrt(m / 2)) * gamma((m - 1) / 2) )

a <- (m * (cm^2)) / (m - 2)

if(ES_index == "g"){

g <- ES_i

}

if(ES_index == "d"){

g <- ES_i * cm

}

tau2_estim_MM <- ( (sum(g^2) - sum(a / nn)) / sum(a) ) -

( sum(a / length(g)) / (length(g) - 1) ) *

(

( ( length(g) * (mean(g)^2) - sum(a / (length(g) * nn)) ) / sum(a / length(g)) ) -

(sum(g^2) - sum(a / nn)) / sum(a)

)

if(tau2_estim_MM > 0){

sigma_g_estim <- (a / nn) + a * ((sum(g^2) - sum(a/nn)) / sum(a)) -

(

(sum(a/length(g)) / (length(g)-1)) *

( ( ( length(g) * mean(g)^2 - sum(a/(length(g)*nn)) ) / sum(a/length(g)) ) -

((sum(g^2) - sum(a/nn)) / sum(a)) )

)

} else {

tau2_estim_MM <- 0

sigma_g_estim <- (1 / nn) + ((1 - (1/a)) * ((sum(nn*g)/sum(nn))^2)) # Hedges (1983) (with ñ weighted delta estimate)

}

var_g <- (1 / nn) + ((1 - (1/a)) * (g^2)) # Hedges (1983)

mu_delta_estim_MM <- sum((1 / sigma_g_estim) * g) / sum(1 / sigma_g_estim)

amp_t <- sqrt((1 / sum(1 / sigma_g_estim))) * qt(0.975, df = length(g)-1)

mu_delta_lb <- mu_delta_estim_MM - amp_t

mu_delta_ub <- mu_delta_estim_MM + amp_t

REML <- rma.uni(yi = g, vi = var_g, method = "REML", test = "t") #Restricted Maximum Likelihood

DL <- rma.uni(yi = g, vi = var_g, method = "DL", test = "t") #Dersimonian-Laird

# Tau2 CI by bootstrap sampling

tau2_boot <- matrix(NA, boot_samples, 3)

colnames(tau2_boot) <- c("tau2_MM_i", "tau2_REML_i", "tau2_DL_i")

for(i in 1:boot_samples){ # bootstrap loop

samp_i <- sample(1:length(g), replace = T)

data_samp_i <- cbind(g, n1, n2, nn, a, var_g)[samp_i,]

colnames(data_samp_i) <- c("g", "n1", "n2", "nn", "a", "var_g")

tau2_MM_i <- ( (sum(data_samp_i[,"g"]^2) - sum(data_samp_i[,"a"] / data_samp_i[,"nn"])) / sum(data_samp_i[,"a"]) ) -

( sum(data_samp_i[,"a"] / nrow(data_samp_i)) / (nrow(data_samp_i) - 1) ) *

(

( ( nrow(data_samp_i) * (mean(data_samp_i[,"g"])^2) - sum(data_samp_i[,"a"] / (nrow(data_samp_i) * data_samp_i[,"nn"])) ) / sum(data_samp_i[,"a"] / nrow(data_samp_i)) ) -

(sum(data_samp_i[,"g"]^2) - sum(data_samp_i[,"a"] / data_samp_i[,"nn"])) / sum(data_samp_i[,"a"])

)

tau2_MM_i <- ifelse(tau2_MM_i > 0, tau2_MM_i, 0)

tau2_REML_i <- tryCatch(

{rma.uni(yi = data_samp_i[,"g"], vi = data_samp_i[,"var_g"], method = "REML", test = "t", control = list(maxiter = 200))$tau2}, #Restricted Maximum Likelihood

error = function(x){return(NA)}

)

tau2_DL_i <- tryCatch(

{rma.uni(yi = data_samp_i[,"g"], vi = data_samp_i[,"var_g"], method = "DL", test = "t", control = list(maxiter = 200))$tau2}, #Dersimonian-Laird

error = function(x){return(NA)}

)

tau2_boot[i,] <- c(tau2_MM_i, tau2_REML_i, tau2_DL_i)

}

tau2_CI_MM <- quantile(tau2_boot[,"tau2_MM_i"], probs = c(0.025, 0.975), na.rm = T)

tau2_CI_REML <- quantile(tau2_boot[,"tau2_REML_i"], probs = c(0.025, 0.975), na.rm = T)

tau2_CI_DL <- quantile(tau2_boot[,"tau2_DL_i"], probs = c(0.025, 0.975), na.rm = T)

res <- c(mu_delta_estim_MM, mu_delta_lb, mu_delta_ub, tau2_estim_MM, tau2_CI_MM,

REML$beta, REML$ci.lb, REML$ci.ub, REML$tau2, tau2_CI_REML,

DL$beta, DL$ci.lb, DL$ci.ub, DL$tau2, tau2_CI_DL)

names(res) <- c("mu_delta_estim_MM", "mu_delta_estim_MM_lb", "mu_delta_estim_MM_ub", "tau2_estim_MM", "tau2_estim_MM_lb", "tau2_estim_MM_ub",

"REML_mu_delta", "REML_mu_delta_lb", "REML_mu_delta_ub", "REML_tau2", "REML_tau2_lb", "REML_tau2_ub",

"DL_mu_delta", "DL_mu_delta_lb", "DL_mu_delta_ub", "DL_tau2", "DL_tau2_lb", "DL_tau2_ub")

return(res)

}

#Reading and preparing the data base (the file name must be "Data.xlsx")

data <- read_excel("Data.xlsx", sheet = 1)

var <- colnames(data)

data <- matrix(as.numeric(unlist(data)), nrow = nrow(data), ncol = ncol(data))

colnames(data) <- var

#Estimation from g (or d), n1 and n2

estim(ES_i = data[,"g"], n1 = data[,"n1"], n2 = data[,"n2"], ES_index = "g") #If the ES index is g

estim(ES_i = data[,"d"], n1 = data[,"n1"], n2 = data[,"n2"], ES_index = "d") #If the ES index is d
